# CBF**β**-SMMHC–driven leukemogenesis requires enhanced RUNX1-DNA binding affinity in mice

**DOI:** 10.1172/JCI192923

**Published:** 2025-08-05

**Authors:** Tao Zhen, Yaqiang Cao, Tongyi Dou, Yun Chen, Guadalupe Lopez, Ana Catarina Menezes, Xufeng Wu, John A. Hammer, Jun Cheng, Lisa Garrett, Stacie Anderson, Martha Kirby, Stephen Wincovitch, Bayu Sisay, Abdel G. Elkahloun, Di Wu, Lucio H. Castilla, Wei Yang, Jiansen Jiang, Keji Zhao, P. Paul Liu

**Affiliations:** 1 Oncogenesis and Development Section, Translational and Functional Genomics Branch, National Human Genome Research Institute (NHGRI), NIH, Bethesda, Maryland, USA.; 2 Laboratory of Epigenome Biology, Systems Biology Center, National Heart, Lung, and Blood Institute (NHLBI), NIH, Bethesda, Maryland, USA.; 3 Laboratory of Membrane Proteins and Structural Biology, NHLBI, NIH, Bethesda, Maryland, USA.; 4 Mechanism of DNA Repair, Replication, and Recombination Section, National Institute of Diabetes and Digestive and Kidney Diseases, NIH, Bethesda, Maryland, USA.; 5 Cell and Developmental Biology Center, NHLBI, NIH, Bethesda, Maryland, USA.; 6 Transgenic Mouse Core,; 7 Flow Cytometry Core,; 8 Advanced Imaging and Analysis Core, and; 9 Microarrays and Single-Cell Genomics Core, NHGRI, NIH, Bethesda, Maryland, USA.; 10 Biophysics Core, NHLBI, NIH, Bethesda, Maryland, USA.; 11 Department of Molecular, Cell and Cancer Biology, Chan Medical School, University of Massachusetts, Worcester, Massachusetts, USA.

**Keywords:** Genetics, Hematology, Leukemias, Mouse models, Transcriptomics

## Abstract

The leukemia fusion gene *CBFB-MYH11* requires RUNX1 for leukemogenesis, but the underlying mechanism is unclear. By in vitro studies, we found that CBFβ-SMMHC, the chimeric protein encoded by *CBFB-MYH11*, could enhance the binding affinity between RUNX1 and its target DNA. Increased RUNX1-DNA binding was also observed in myeloid progenitor cells from mice expressing CBFβ-SMMHC. Moreover, only CBFβ-SMMHC variants able to enhance the DNA binding affinity by RUNX1 could induce leukemia in mouse models. Marked transcriptomic changes, affecting genes associated with inflammatory response and target genes of CBFA2T3, were observed in mice expressing leukemogenic CBFβ-SMMHC variants. Finally, we show that CBFβ-SMMHC could not induce leukemia in mice with a *Runx1*-R188Q mutation, which reduces RUNX1 DNA binding but does not affect its interaction with CBFβ-SMMHC or its sequestration to cytoplasm by CBFβ-SMMHC. Our data suggest that, in addition to binding RUNX1 to regulate gene expression, enhancing RUNX1 binding affinity to its target DNA is an important mechanism by which CBFβ-SMMHC contributes to leukemogenesis, highlighting RUNX1-DNA interaction as a potential therapeutic target in inv(16) acute myeloid leukemia.

## Abstract 2

Increased binding affinity between transcription factor RUNX1 and its DNA target sequences is a critical mechanism for the fusion gene CBFB-MYH11 to induce leukemia.

## Graphical abstract

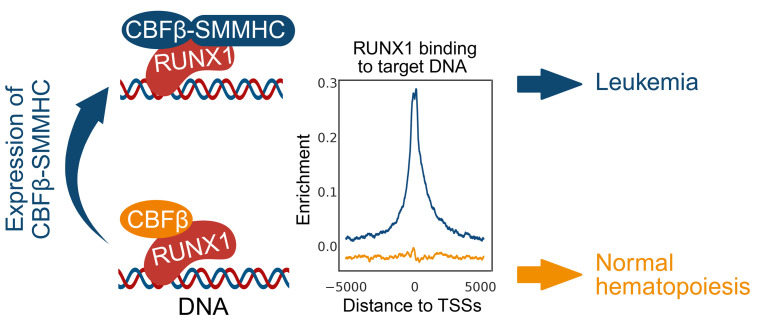

## Introduction

Inversion of chromosome 16 [inv(16)], which is associated with acute myeloid leukemia (AML) subtype M4 with eosinophilia, generates the *CBFB-MYH11* fusion gene (1). CBFβ-SMMHC, encoded by *CBFB-MYH11*, encompasses most of core-binding factor β (CBFβ; 1–165 aa of total 187 aa) and the C-terminal coiled-coil domain of the smooth muscle myosin heavy chain (SMMHC) (2, 3). Previous studies have indicated that *CBFβ-MYH11* is necessary, but not sufficient, for leukemogenesis (4, 5).

CBF binds with the key hematopoietic transcription factor RUNX1, which is one of the three α subunits in the core-binding factor family (RUNX1, RUNX2, and RUNX3), leading to the stabilization of the RUNX-DNA interaction and gene expression regulation through the transactivation domain of RUNX proteins (6, 7). Earlier data in cultured cells showed that CBFβ-SMMHC could sequester RUNX1 in the cytoplasm, which may prevent it from regulating target genes in the nucleus (8–10). In the *Cbfb-MYH11*–knockin (*Cbfb^+/MYH11^*) mouse embryos, definitive hematopoiesis was blocked at embryonic day 12.5 (E12.5), accompanied by central nervous system hemorrhage and lethality at E13.5 (11), a phenotype shared with *Runx1*-null (*Runx1^–/–^*) and *Cbfb*-null (*Cbfb^–/–^*) embryos (12–16). These in vitro and in vivo findings seem to suggest that CBFβ-SMMHC may initiate leukemogenesis through dominant repression of RUNX1 and CBFβ functions.

However, we later found that the relationship between RUNX1 and CBFβ-SMMHC is more complicated, and reduced Runx1 activity could actually rescue some of the differentiation defects during embryonic hematopoiesis and, unexpectedly, delay leukemogenesis in mice (17). More recently, we demonstrated that RUNX1 is required for the leukemia-initiating cell population (abnormal myeloid progenitor [AMP]) in mice with conditional knockin of *Cbfb-MYH11* (18). Moreover, our data suggested that RUNX1 and CBFβ-SMMHC may work together to regulate RUNX1 target genes (18). Importantly, mice with conditional knockin of *Cbfb-MYH11* over *Runx1*-null background did not develop leukemia, suggesting that *Runx1* is indispensable for leukemogenesis by *Cbfb-MYH11*.

The mechanism for the RUNX1 requirement in leukemogenesis by CBFβ-SMMHC is still unclear. CBFβ-SMMHC retains the RUNX-binding domain from CBFβ and also contains a second RUNX high-affinity binding domain (HABD) in the SMMHC tail, resulting in a higher binding affinity for RUNX1 than wild-type CBFβ (19). However, chimeric mice expressing CBFβ-SMMHC with deletion of the HABD (CBFβ-SMMHC-ΔHABD) only partially rescued the hematopoiesis phenotype, and unexpectedly accelerated leukemia development caused by CBFβ-SMMHC, challenging the importance of RUNX1 for CBFβ-SMMHC–mediated leukemogenesis.

Another important domain is the C-terminal coiled-coil region of SMMHC, which interacts with mSin3A to repress transcription, and catalyzes homodimerization and multimerization of the CBFβ-SMMHC fusion protein (8, 10, 20, 21). To determine the role of this coiled-coil region for leukemogenesis in vivo, we generated mice expressing knocked-in CBFβ-SMMHC with a 95–amino acid C-terminal truncation (including the coiled-coil region), and these mice failed to develop leukemia (22). In addition, mice expressing CBFβ-SMMHC with missense mutations in the C-terminal multimerization domain (mDE), which impairs the multimerization but leaves the transcriptional repression domain intact (20), did not develop leukemia (23). The results suggested that the C-terminal domain, especially the multimerization domain, is important for CBFβ-SMMHC to induce leukemia.

To unravel the underlying mechanism of RUNX1’s indispensable role in *CBFB-MYH11*–induced leukemia (18), we conducted an integrated analysis of the leukemogenicity of *CBFB-MYH11* and two *CBFB-MYH11* mutations, deletion of the RUNX1 high-affinity binding domain (*CBFB-MYH11-ΔHABD*) and mutation in the C-terminal multimerization domain (*CBFB-MYH11-mDE*), and investigated their effects on RUNX1’s functions. We firstly generated conditional *Cbfb-MYH11-ΔHABD* knockin mice to overcome the limitations of the previous chimeric mouse model, and we found that the conditional *Cbfb-MYH11-ΔHABD* knockin mice exhibited a leukemia profile similar to that of *Cbfb-MYH11* mice albeit with longer latency. We then focused on how the ΔHABD and mDE mutations influence the interaction between CBFβ-SMMHC and RUNX1, as well as the binding of RUNX1 to its target DNA. We found that leukemogenic CBFβ-SMMHC proteins (CBFβ-SMMHC and CBFβ-SMMHC-ΔHABD) enhance the binding affinity of RUNX1 to target oligonucleotides both in bio-layer interferometry (BLI) assay with purified proteins, and also in chromatin immunocleavage sequencing (ChIC-Seq) assay with myeloid progenitor cells from mice expressing CBFβ-SMMHC proteins. Importantly, we provide direct evidence that RUNX1 binding to target DNA is critical for leukemogenesis by CBFβ-SMMHC, since CBFβ-SMMHC could not induce leukemia in mice with a heterozygous *Runx1*-R188Q mutation. Through RNA-Seq we further showed that the AMP population in the *Runx1^+/R188Q^Mx1-CreCbfb^+/56M^* mice had a very different gene expression profile from that of the AMP population in the *Mx1-CreCbfb^+/56M^* mice. Additionally, by comparing the gene expression profiles of mice expressing CBFβ-SMMHC or related mutants, we identified marked transcriptomic changes associated with leukemogenic CBFβ-SMMHC variants, which include genes associated with inflammatory response.

## Results

### Conditional Cbfb-MYH11-ΔHABD knockin mice exhibit a leukemia profile similar to that of Cbfb-MYH11 mice.

To investigate the underlying mechanism of RUNX1’s indispensable role in *CBFB-MYH11*–induced leukemia (18), we conducted an integrated analysis of *Cbfb-MYH11* and two *Cbfb-MYH11* mutants, *Cbfb-MYH11-ΔHABD* and *Cbfb-MYH11-mDE* (as illustrated in Supplemental Figure 1; supplemental material available online with this article; https://doi.org/10.1172/JCI192923DS1), regarding their effects on RUNX1 function. As we have reported previously, *Cbfb-MYH11-mDE* (23) was unable to induce leukemia, whereas *CBFB-MYH11-ΔHABD* unexpectedly accelerated leukemia development (24). The latter finding appears inconsistent with our most recent observation, which highlights the essential role of RUNX1 in *Cbfb-MYH11*–induced leukemogenesis (18), probably owing to limitations associated with the chimeric mouse model for *CBFB-MYH11-ΔHABD*.

**Figure 1:**
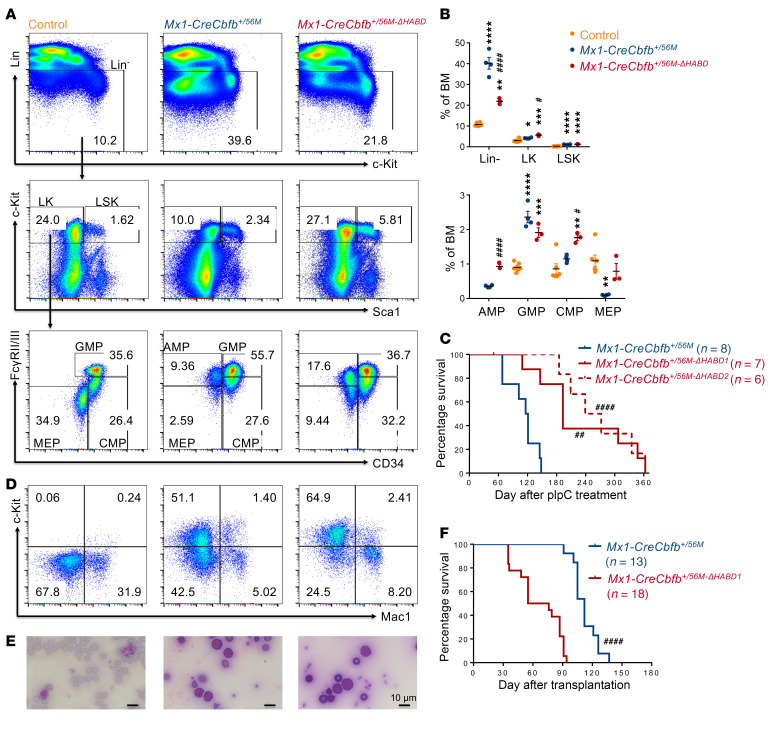
Conditional knockin of *Cbfb-MYH11-ΔHABD* in mice reveals a leukemia profile similar to that of the conditional *Cbfb-MYH11* knockin model. (**A**–**E**) Mice of indicated genotypes were treated with pIpC to induce the expression of *Cbfb-MYH11* or *Cbfb-MYH11-ΔHABD*. (**A**) Representative FACS plots of bone marrow cells from mice treated with pIpC for 2–3 weeks and gated on single cells (top plots), Lin^–^ cells (middle plots), and LK cells (bottom plots). *N* = 3–6 mice per genotype. (**B**) Dot plot showing the percentages (mean ± SEM) of populations in bone marrow cells analyzed by flow cytometry, as presented in **A**, with each dot representing an individual sample. Statistical significance was assessed using 2-tailed Student’s *t* test for comparing between 2 groups (AMP population) and 1-way ANOVA followed by Tukey’s post hoc test for comparisons among multiple groups (all other populations except AMP). (**C**) Kaplan-Meier survival curves of these mice are shown. Two independent *Cbfb^+/56M-ΔHABD^* lines, *Cbfb^+/56M-ΔHABD1^* and *Cbfb^+/56M-ΔHABD2^*, are shown. Statistical significance was assessed using the log-rank test. (**D** and **E**) Representative flow cytometry plots showing cells in the peripheral blood (**D**) and Wright-Giemsa–stained peripheral blood smears (**E**) of end-stage *Mx1-CreCbfb^+/56M^* and *Mx1-CreCbfb^+/56M-ΔHABD^* mice. *N* = 7–10 mice per genotype. Scale bars: 10 μm for all panels. (**F**) Kaplan-Meier survival curves of sublethally irradiated (650 rad) C57BL/6-CD45.1 × 129/SvEv F_1_ mice that underwent transplantation of 1 million spleen cells from end-stage *Mx1-CreCbfb^+/56M^* and *Mx1-CreCbfb^+/56M-ΔHABD^* mice. Statistical significance was assessed using the log-rank test. *P < 0.05, **P < 0.01, ***P < 0.001, ****P < 0.0001, each compared with the control. ^#^*P* < 0.05, ^##^*P* < 0.01, ^####^*P* < 0.0001, each compared with the *Mx1-CreCbfb^+/56M^* group.

To definitively address the leukemogenicity of *Cbfb-MYH11-ΔHABD* and limitations associated with chimeric mouse models, we generated a conditional *Cbfb-MYH11-ΔHABD* knockin mouse model (*Cbfb^+/56M-ΔHABD^*) by deleting nucleotides 44–166 of *MYH11* (HABD domain, nucleotides 40–168) in the conditional *Cbfb-MYH11* knockin allele (25) using a CRISPR/Cas9 gene editing strategy (Supplemental Figure 2A). We then crossed *Cbfb^+/56M-ΔHABD^* mice with *Mx1*-*Cre* mice (26) to generate *Mx1-CreCbfb^+/56M-ΔHABD^* mice. Adult mice (8–24 weeks old), including *Mx1-CreCbfb^+/56M-ΔHABD^*, *Mx1-CreCbfb^+/56M^*, and their littermate controls (wild type; *Mx1-Cre* or other genotypes without *Mx1-Cre*), were treated with poly(I:C) (pIpC) to induce *Mx1-Cre* expression. Two to three weeks after pIpC treatment, CBFβ-SMMHC-ΔHABD protein was expressed in the bone marrow of the *Mx1-CreCbfb^+/56M-ΔHABD^* mice, with expression levels comparable to those of CBFβ-SMMHC in *Mx1-CreCbfb^+/56M^* mice (Supplemental Figure 2B). Additionally, expression of *Cbfb-MYH11-ΔHABD* in adult mice led to aberrant hematopoiesis, similar to mice expressing *Cbfb-MYH11*, including an increase in lineage-negative (Lin^–^) and myeloid progenitor cells (LK; Lin^–^c-Kit^+^Sca1^–^) in bone marrow, along with the presence of abnormal myeloid progenitor cells (AMP; LK/CD34^–^FcγRII/III^+^) (25, 27) (Figure 1, A and B).

We also monitored mice for leukemia after pIpC treatment. As expected, *Mx1-CreCbfb^+/56M^* mice died from AML with a median survival of 118.5 days (Figure 1C). All *Mx1-CreCbfb^+/56M-ΔHABD^* mice also died from AML with a longer median survival of 193 days (line *Cbfb^+/56M-ΔHABD1^*, referred to as *CreCbfb^+/56M-ΔHABD^*) and 255.5 days (line *Cbfb^+/56M-ΔHABD2^*). In addition, similar leukemia was developed in these 2 mouse models, as demonstrated by the complete blood count (Supplemental Figure 2C), the expression of cell surface markers on leukemic cells from peripheral blood, spleen, and bone marrow (Figure 1D and Supplemental Figure 2D), and the morphology of leukemic cells in peripheral blood (Figure 1E). Furthermore, transplantation of spleen cells from both leukemic mice induced similar leukemia in recipients, as evidenced by the expression of similar cell surface markers in peripheral blood, spleen, and bone marrow of end-stage recipients (Supplemental Figure 2E). Surprisingly, transplantation of spleen cells from *Mx1-CreCbfb^+/56M-ΔHABD^* leukemic mice led to leukemia with shorter latency in the recipient mice than transplantation of spleen cells from *Mx1-CreCbfb^+/56M^* mice (Figure 1F). Given that deletion of the HABD domain from CBFβ-SMMHC reduces its binding affinity for RUNX1 (24), these findings suggest that the attenuated interaction between RUNX1 and CBFβ-SMMHC leads to delayed leukemia development but does not impact the overall leukemogenic potential of the fusion protein.

### Leukemogenic CBFβ-SMMHC proteins enhance RUNX1 Runt homology domain binding affinity to the target oligonucleotide.

**Figure 2:**
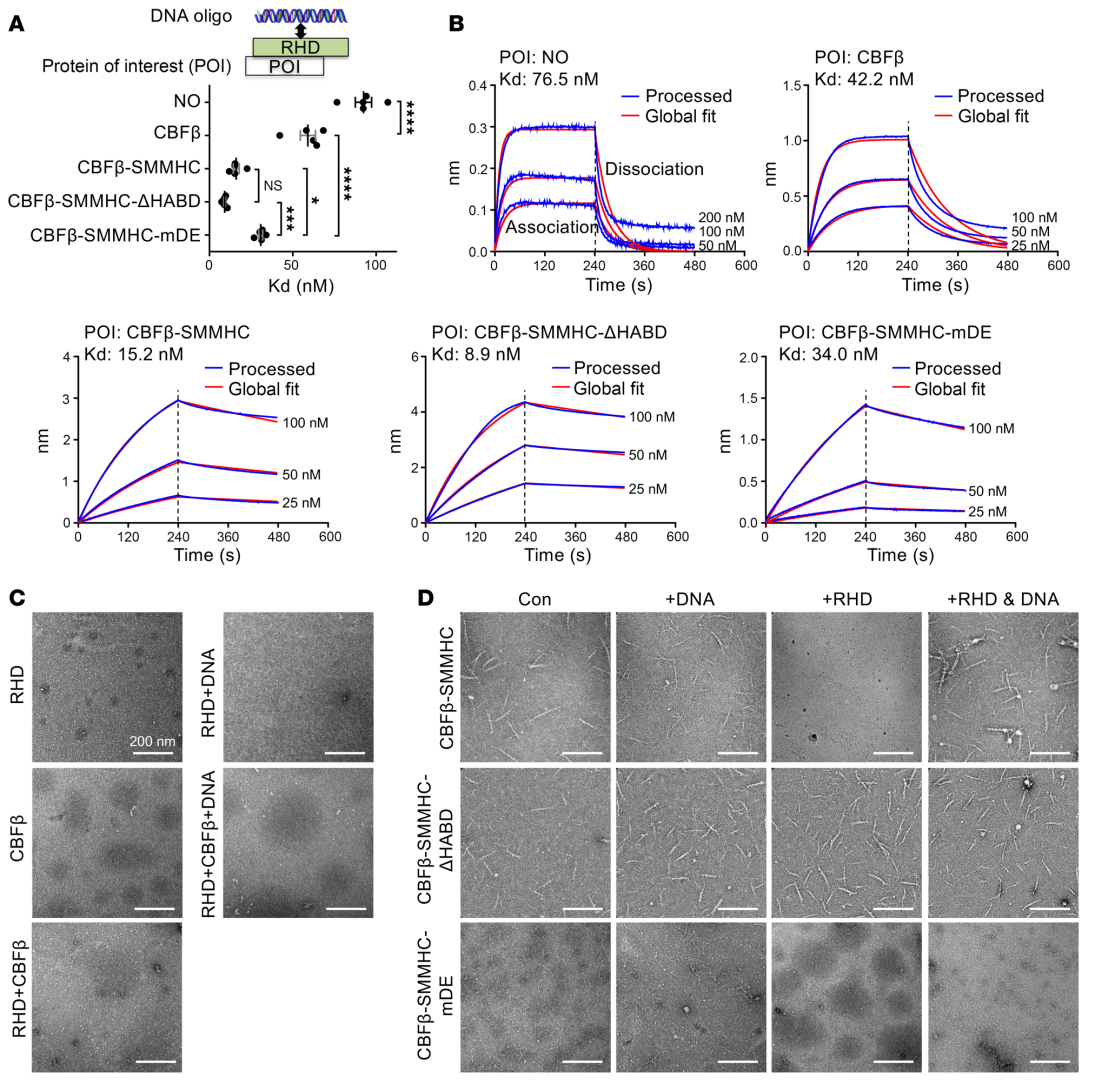
Leukemogenic CBFβ-SMMHC proteins enhance RUNX1 RHD binding affinity to target DNA sequences. (**A** and **B**) BLI assay to evaluate the interaction between the RHD and its target DNA in the presence of the indicated protein of interest. (**A**) Dot plot showing the binding affinities (mean ± SEM) between the RHD and its target DNA for each condition, with each dot representing an individual replicate. Statistical significance was assessed using 1-way ANOVA followed by Tukey’s post hoc test. *P < 0.05, ***P < 0.001, ****P < 0.0001. (**B**) Representative binding curves from the BLI assay, showing both the association and dissociation phases. (**C** and **D**) Representative images of the indicated proteins or protein-DNA complexes as detected by negative stain electron microscopy. Images shown are representative of 2–4 independent experiments. Scale bars: 200 nm for all panels.

We then explored the effects of these two CBFβ-SMMHC mutations, CBFβ-SMMHC-ΔHABD and CBFβ-SMMHC-mDE, on RUNX1 function, focusing on how these mutations influence CBFβ-SMMHC binding affinity for RUNX1 and RUNX1 binding to its target DNA (28). We conducted BLI experiments using proteins purified from *E*. *coli* with relevant plasmid constructs. We were unable to purify the full-length RUNX1 protein; instead, we used the purified runt homology domain (RHD), as it is responsible for interacting with both CBFβ and DNA. Using BLI assay, as expected, deletion of the HABD domain (CBFβ-SMMHC-ΔHABD) significantly reduced the binding affinity of CBFβ-SMMHC to RHD (19) (Supplemental Figure 3, A and B). On the other hand, mutations in the multimerization domain (CBFβ-SMMHC-mDE) did not impact CBFβ-SMMHC’s binding affinity to RHD (Supplemental Figure 3, A and B). These results suggested that the binding affinity of CBFβ-SMMHC for the RHD (RUNX1) is not a determining factor for leukemogenesis, since mice expressing *Cbfb-MYH11-ΔHABD* could develop leukemia while mice expressing *Cbfb-MYH11-mDE* could not. In addition, we examined the interaction between RHD and its target DNA oligonucleotide in the presence of CBFβ-SMMHC or related mutants. We selected the DNA target oligonucleotide that has been previously used to determine the structure of the RHD, CBFβ, and DNA (28). We found that the binding affinity between RHD and its target DNA oligonucleotide was significantly enhanced in the presence of CBFβ-SMMHC and CBFβ-SMMHC-ΔHABD (which can induce leukemia), but not of CBFβ-SMMHC-mDE (which cannot induce leukemia) (Figure 2, A and B). These findings suggest that the enhanced binding affinity between RUNX1 and its target DNA, rather than enhanced binding affinity between RUNX1 and CBFβ-SMMHC, is important for leukemogenesis by CBFβ-SMMHC.

We also performed negative staining to evaluate the effect of the CBFβ-SMMHC mutations on filament formation, given the filament-forming properties of SMMHC (29). As expected, RHD, CBFβ, and RUNX1 target DNA oligonucleotide alone, or in combination, did not form visible structures (Figure 2C). However, both CBFβ-SMMHC and CBFβ-SMMHC-ΔHABD, but not CBFβ-SMMHC-mDE, were able to form filament structures, alone or in the presence of the DNA oligonucleotide (Figure 2D). Interestingly, RHD binding inhibited filament formation by CBFβ-SMMHC, and this effect was overcome when RUNX1 target DNA oligonucleotide was added. In contrast, RHD could not inhibit filament formation by CBFβ-SMMHC-ΔHABD (Figure 2D), suggesting that HABD interaction is required for RHD to disrupt filament formation by CBFβ-SMMHC, and that binding of target DNA oligonucleotide can reverse this effect. Supplemental Figure 4 presents a hypothetical model to explain these findings. Specifically, certain mutations or interactions alter the structural integrity of the SMMHC region, potentially disrupting its ability to form filaments. These results suggested that the ability to form filament, with or without RHD and target oligonucleotide, is important for leukemogenesis by CBFβ-SMMHC, and that filament formation by CBFβ-SMMHC may contribute to enhanced DNA binding affinity. The findings are consistent with our previous publications in mouse models demonstrating that the SMMHC multimerization domain, which is responsible for filament formation, is required for leukemogenesis (22, 23).

### Leukemogenic CBFβ-SMMHC proteins increase RUNX1’s DNA binding in vivo.

**Figure 3:**
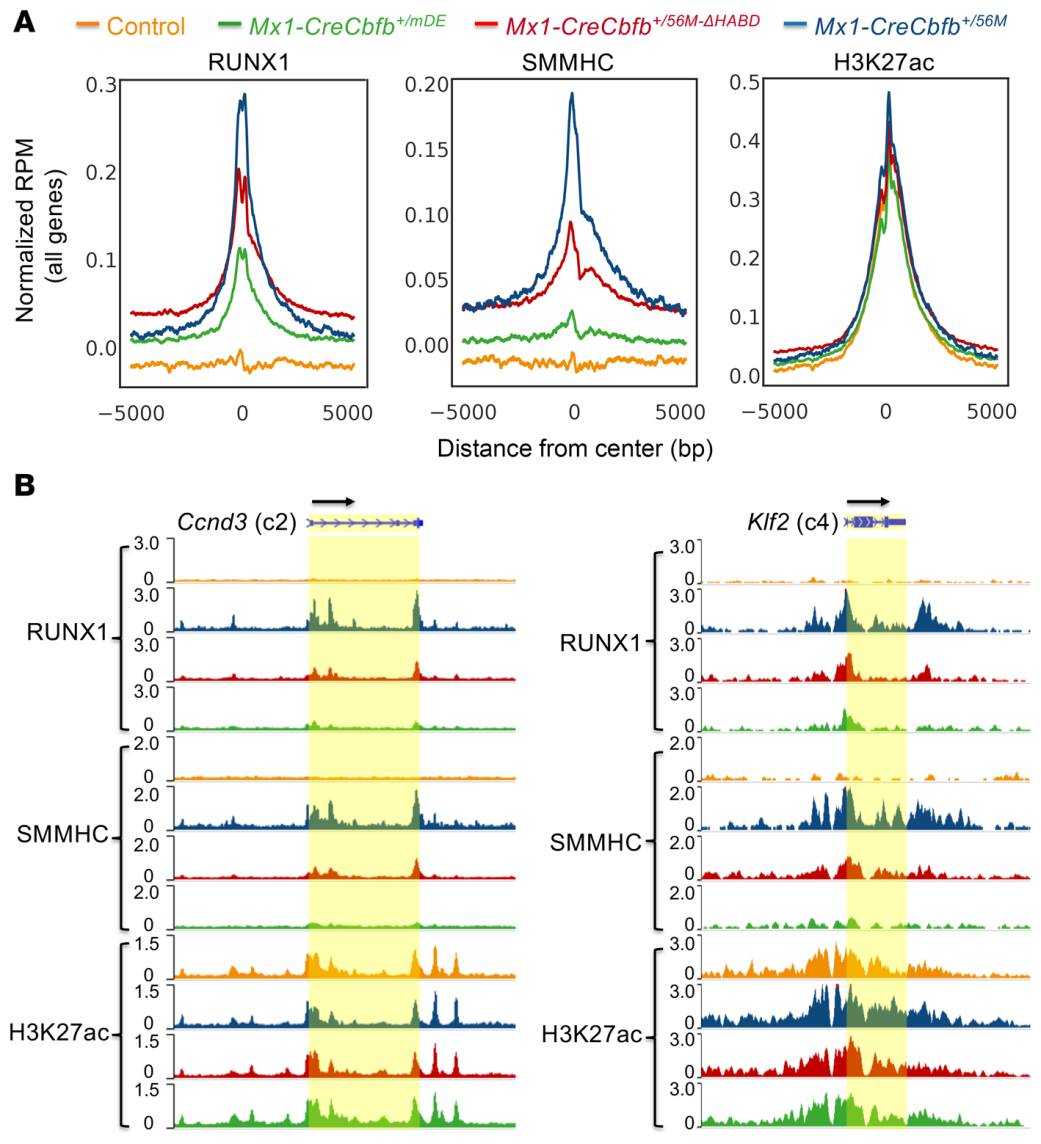
Leukemogenic CBFβ-SMMHC proteins enhance RUNX1 binding enrichment at transcription start sites in murine LK cells. (**A** and **B**) ChIC-Seq was performed on LK cells isolated from control, *Mx1-CreCbfb^+/56M^*, *Mx1-CreCbfb^+/56M-ΔHABD^*, and *Mx1-CreCbfb^+/mDE^* mice 2–3 weeks after pIpC treatment. (**A**) Aggregated average ChIC-Seq binding profile of indicated proteins at the transcription start sites (TSSs) of all the genes, normalized with IgG signals subtracted for each condition. The center locations in the graphs are the TSSs. RPM, reads per million mapped reads. (**B**) Genome browser images displaying ChIC-Seq signals at the *Ccnd3* and *Klf2* gene loci with RUNX1, SMMHC, and H3K27ac antibodies, respectively, with the transcription direction indicated by black arrows. ChIC-Seq tracks are color-coded to match the corresponding mouse genotypes shown in **A**.

We then wanted to explore the effect of CBFβ-SMMHC or related mutants on RUNX1’s binding to its target DNA in vivo by performing ChIC-Seq assays (18, 30) on the myeloid progenitor cells (LK; Lin^–^c-Kit^+^Sca1^–^) isolated from control (wild-type), *Mx1-CreCbfb^+/56M^*, *Mx1-CreCbfb^+/56M-ΔHABD^*, and *Mx1-CreCbfb^+/mDE^* mice with RUNX1, SMMHC, and H3K27ac antibodies. As SMMHC is not expressed in hematopoietic cells (Human Protein Atlas), the SMMHC antibody allows specific identification of CBFβ-SMMHC. As expected, negligible binding of SMMHC was detected in control samples, as indicated by the lack of SMMHC binding enrichment at transcription start sites (TSSs) (Figure 3, A and B). SMMHC binding enrichment at TSSs was also very low in *Mx1-CreCbfb^+/mDE^* samples, indicating that monomeric CBFβ-SMMHC proteins have low binding enrichment. In contrast, SMMHC binding enrichment at TSSs was substantially higher in *Mx1-CreCbfb^+/56M^* and *Mx1-CreCbfb^+/56M-ΔHABD^* cells compared with control or *Mx1-CreCbfb^+/mDE^* cells (Figure 3, A and B). Furthermore, SMMHC binding enrichment at TSSs was lower in *Mx1-CreCbfb^+/56M-ΔHABD^* cells compared with *Mx1-CreCbfb^+/56M^* cells, which may have contributed to delayed leukemia development in *Mx1-CreCbfb^+/56M-ΔHABD^* mice. Similar findings were noted for RUNX1 binding enrichment at TSSs among these samples, while no obvious difference was observed in terms of the enrichment of H3K27ac at TSSs (Figure 3, A and B). RUNX1 binding enrichment at TSSs in control/wild-type mice is detectable at many loci, but at a very low level in LK cells, suggesting low RUNX1 binding in the absence of CBFβ-SMMHC. Moreover, in the human inv(16) ME-1 cell line (31), RUNX1 binding enrichment at TSSs was reduced following CBFB-MYH11 knockdown (Supplemental Figure 5). Although a notable enrichment of RUNX1 at TSSs was still observed in the knockdown cells, this is likely due to incomplete knockdown of *CBFB-MYH11*, as a limited number of differentially expressed genes (DEGs) were detected upon *CBFB-MYH11* knockdown (31). These data further support the conclusion that CBFβ-SMMHC enhances RUNX1’s DNA binding ability in inv(16)-positive cells. Consistently, mutations in the RUNX1 gene, particularly those affecting its DNA-binding domain, have been associated with various hematological malignancies, but notably absent in t(8;21) and inv(16) AML (32–34). These results suggested that both CBFβ-SMMHC and CBFβ-SMMHC-ΔHABD increased RUNX1 DNA binding in hematopoietic cells, and that binding of RUNX1/CBFβ-SMMHC to DNA and direct transcription regulation are critical for CBFβ-SMMHC to induce leukemia.

### Leukemogenic CBFβ-SMMHC variants and RUNX1 directly regulate target genes, leading to marked transcriptome changes in LK cells.

**Figure 4:**
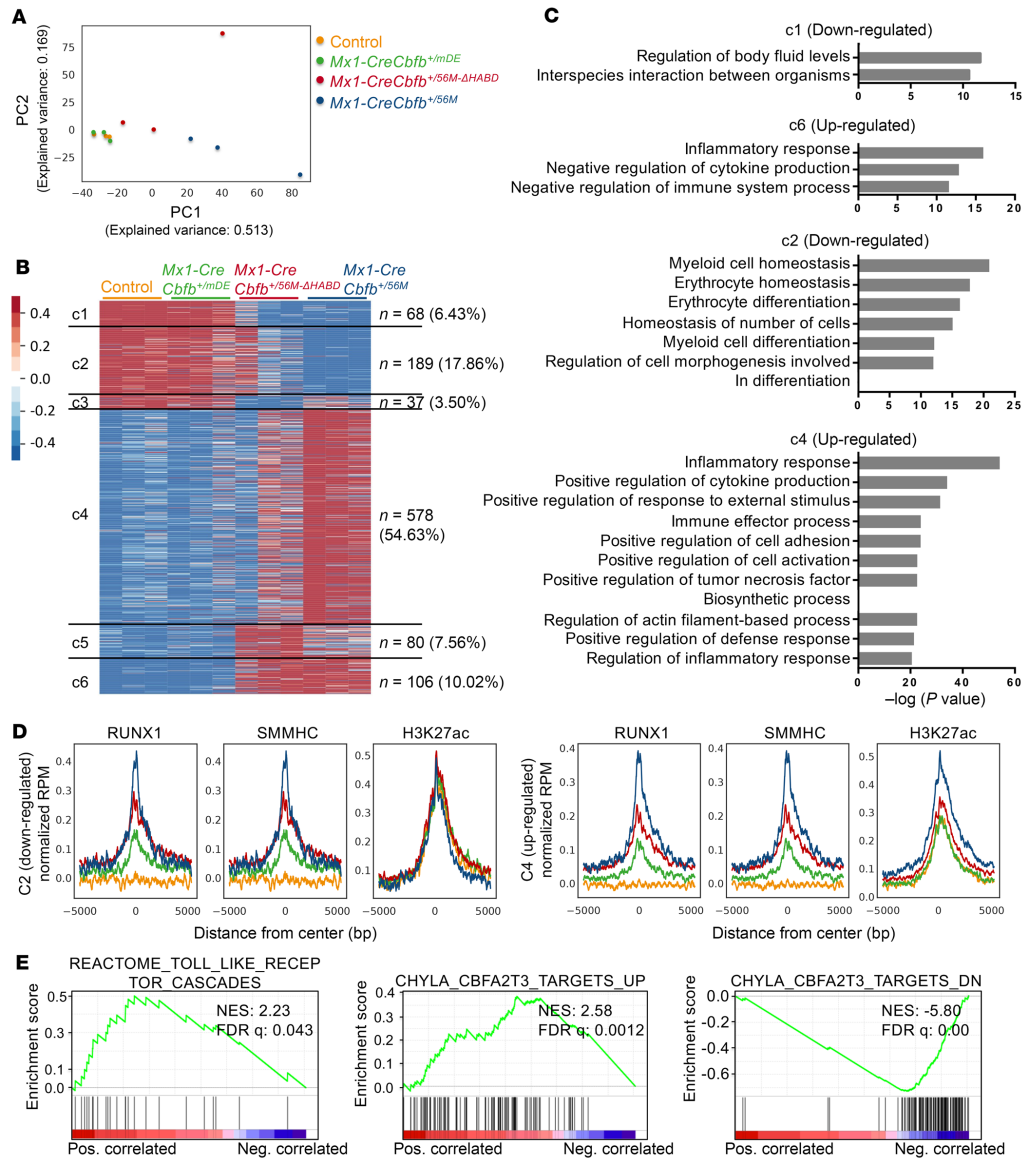
Leukemogenic CBFβ-SMMHC variants and RUNX1 directly regulate target genes, driving marked transcriptome changes in LK cells. (**A**–**C**) RNA-Seq was performed on LK cells isolated from control, *Mx1-CreCbfb^+/56M^*, *Mx1-CreCbfb^+/56M-ΔHABD^*, and *Mx1-CreCbfb^+/mDE^* mice 2–3 weeks after pIpC treatment. *N* = 3 for each genotype. (**A**) Two-dimensional principal component analysis plot illustrating the variation across these samples. (**B**) Heatmap representation of DEGs in LK cells among control, *Mx1-CreCbfb*^+/56M-*Δ*HABD^, and *Mx1-CreCbfb^+/56M^* mice, with corresponding expression level in *Mx1-CreCbfb^+/mDE^* mice also included. Numbers and percentages of DEGs in each cluster are listed on the right. (**C**) Gene Ontology analysis of the DEGs in clusters c1, c2, c4, and c6, as shown in **B**. Only terms with –log(*P* value) greater than 10 are shown. (**D**) Average ChIC-Seq binding profiles at the TSSs of genes downregulated in cluster c2 and upregulated in c4, respectively. Lines are color-coded to match the corresponding mouse genotypes shown in **A**. (**E**) GSEA (pre-ranked) identified curated gene sets significantly enriched in the DEGs shown in **B**.

Given the variation in leukemogenic potential among CBFβ-SMMHC and its related mutants, we performed RNA sequencing on LK cells isolated from control, *Mx1-CreCbfb^+/56M^*, *Mx1-CreCbfb^+/56M-ΔHABD^*, and *Mx1-CreCbfb^+/mDE^* mice (2–3 weeks after pIpC treatment) and then integrated gene expression profiles with the leukemogenic potential to identify genes and pathways responsible for CBFβ-SMMHC–induced leukemia development. Principal component analysis (PCA) revealed that LK cells from control and *Mx1-CreCbfb^+/mDE^* mice, which do not develop leukemia, clustered together, and were distinctly separated from LK cells from *Mx1-CreCbfb^+/56M^* and *Mx1-CreCbfb^+/56M-ΔHABD^* mice, which do develop leukemia (Figure 4A). In comparison with genes in the control mice, we did not identify any DEGs with *q* value less than 0.01 and absolute fold change ≥ 2 in *Mx1-CreCbfb^+/mDE^* mice, indicating a high degree of similarity between these 2 groups of mice (Supplemental Figure 6A and Supplemental Table 2). We then performed pairwise comparisons among the control, *Mx1-CreCbfb^+/56M^*, and *Mx1-CreCbfb^+/56M-ΔHABD^* mice to identify DEGs. The DEGs are visualized in a heatmap (Figure 4B) and listed in Supplemental Table 3. The heatmap revealed 6 distinct clusters of DEGs, with 294 genes (27.79%, clusters c1, c2, and c3) downregulated and 764 genes (72.21%, clusters c4, c5, and c6) upregulated in *Mx1-CreCbfb^+/56M^* or *Mx1-CreCbfb^+/56M-ΔHABD^* mice compared with control mice (Figure 4B). Interestingly, the majority of these DEGs (72.49%, clusters c2 and c4) showed attenuated expression changes in *Mx1-CreCbfb^+/56M-ΔHABD^* mice compared with *Mx1-CreCbfb^+/56M^* mice; a smaller portion of these DEGs (16.45%, clusters c1 and c6) were similarly down- or upregulated in both *Mx1-CreCbfb^+/56M^* and *Mx1-CreCbfb^+/56M-ΔHABD^* mice compared with the control mice; only a minor fraction of DEGs (11.06%, clusters c3 and c5) were uniquely down- or upregulated in *Mx1-CreCbfb^+/56M-ΔHABD^* mice. These findings suggest that deletion of the HABD domain primarily affects the magnitude, rather than the direction, of gene expression changes mediated by CBFβ-SMMHC (Figure 4B and Supplemental Figure 6B). Gene Ontology analysis showed that DEGs in cluster c2 (downregulated) were associated with the terms related to myeloid cell homeostasis and differentiation, and DEGs in clusters c4 and c6 (upregulated) were associated with several terms related to inflammatory response (Figure 4C). In addition, DEGs in cluster c1 (downregulated) were associated with the term related to regulation of body fluid levels (Figure 4C), and DEGs in clusters c3 and c5 were associated with fewer significant terms, such as “cellular divalent inorganic cation homeostasis” and “negative regulation of phosphorylation,” respectively (Supplemental Figure 6C). These results suggested that the full-length CBFβ-SMMHC and CBFβ-SMMHC-ΔHABD block normal hematopoiesis and upregulate inflammatory responses, which may contribute to leukemia development in *Mx1-CreCbfb^+/56M^* and *Mx1-CreCbfb^+/56M-ΔHABD^* mice.

We then investigated how RUNX1 and CBFβ-SMMHC regulate the expression of DEGs in those clusters by examining their binding enrichment at the TSSs of the DEGs within each cluster. Similar to the pattern observed for all genes (Figure 3A), binding enrichment for both RUNX1 and SMMHC at TSSs for DEGs in all clusters was higher in *Mx1-CreCbfb^+/56M^* and *Mx1-CreCbfb^+/56M-ΔHABD^* mice, compared with that observed in *Mx1-CreCbfb^+/mDE^* and control mice (Figure 3B, Figure 4D, and Supplemental Figure 6, D–F), suggesting that many of the genes within these DEGs are direct targets of RUNX1 and CBFβ-SMMHC. Notably, most of these DEGs were upregulated in *Mx1-CreCbfb^+/56M^* and *Mx1-CreCbfb^+/56M-ΔHABD^* mice (clusters c4, c5, and c6 compared with clusters c1, c2, and c3), consistent with our previous finding that the transcriptional activation role of CBFβ-SMMHC is more prominent in *Cbfb-MYH11*–induced leukemia (18). We also integrated ChIC-Seq and RNA-Seq data to identify RUNX1-bound DEGs potentially critical for leukemogenesis. As shown in Supplemental Table 3 and Figure 3, genes such as *Ccnd3* and *Klf2* emerged as candidate effectors. While these findings provide valuable leads, further studies will be needed to validate their functional roles. In addition, increased H3K27ac modification was exclusively observed at TSSs of upregulated DEGs in clusters c4, c5, and c6 in *Mx1-CreCbfb^+/56M^* and *Mx1-CreCbfb^+/56M-ΔHABD^* mice compared with control and *Mx1-CreCbfb^+/mDE^* mice (Figure 4D and Supplemental Figure 6, D and F), suggesting that H3K27ac modifications correlated with upregulated DEGs in clusters c4 and c6 but not with downregulated DEGs.

We also performed gene set enrichment analysis (GSEA) (35) of the DEGs identified in Figure 4B. Several curated gene sets were significantly enriched. As shown in Figure 4E and Supplemental Table 4, the upregulated DEGs in *Mx1-CreCbfb^+/56M^* mice were positively correlated with several sets related to immune response, such as REACTOME_TOLL_LIKE_RECEPTOR_CASCADES, consistent with the finding that RUNX1 can negatively regulate inflammatory cytokine production (36). Additionally, the upregulated DEGs in *Mx1-CreCbfb^+/56M^* cells also positively correlated with CHYLA_CBFA2T3_TARGETS_UP and negatively correlated with CHYLA_CBFA2T3_TARGETS_DN, suggesting a critical role for CBFA2T3 during leukemia initiation in *Mx1-CreCbfb^+/56M^* mice.

### DNA binding by RUNX1 drives CBFβ-SMMHC–induced leukemia in mice.

It has been reported that human RUNX1b-R174Q mutation (corresponding to the R201Q mutation in human RUNX1c, R188Q in mouse RUNX1c, and R174Q in mouse RUNX1b) significantly decreases its DNA binding capability while preserving its interaction with CBFβ (37). We confirmed that RUNX1c-R201Q was unable to interact with target DNA oligonucleotide using EMSA (Supplemental Figure 7A). Additionally, immunofluorescence assays demonstrated that mouse RUNX1b (mRUNX1b)-R174Q could interact with CBFβ-SMMHC, as mRUNX1b-R174Q was sequestered in the cytoplasm and colocalized with CBFβ-SMMHC in cells cotransfected with both proteins (see below). Consistently, unlike wild-type RUNX1, RUNX1c-R201Q failed to transactivate *CSF1R* luciferase activity in a report assay (38), whether in the absence or presence of CBFβ, CBFβ-SMMHC, or related mutants (Supplemental Figure 7, B and C).

**Figure 5:**
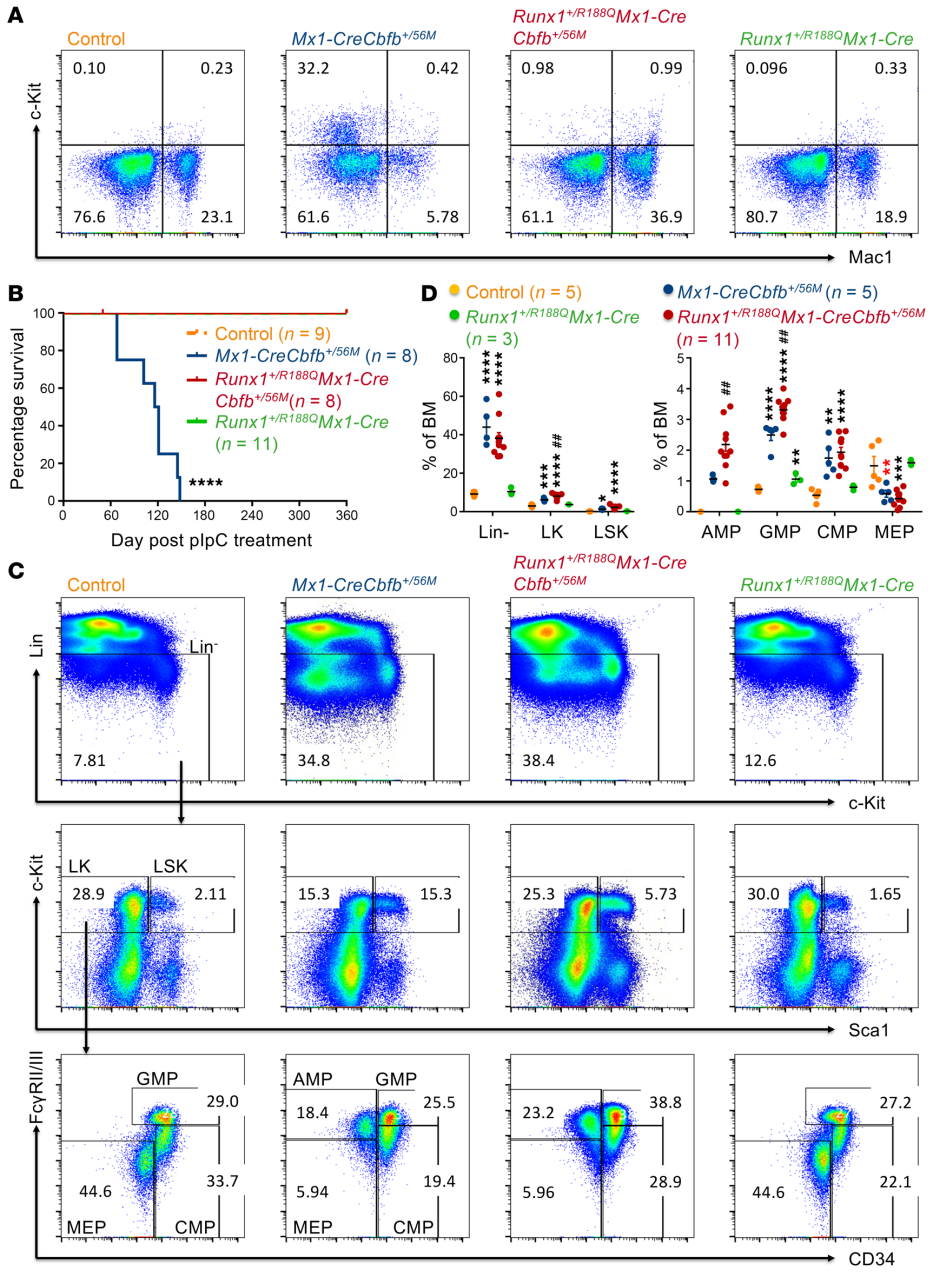
DNA binding by RUNX1 is essential for CBFβ-SMMHC–induced leukemia in mice. (**A**–**D**) Mice of indicated genotypes were treated with pIpC to induce the expression of *Cbfb-MYH11*. (**A**) Representative flow cytometry plots showing cells in the peripheral blood, 11 weeks after pIpC treatment. *N* = 5–8 mice per genotype. (**B**) Kaplan-Meier survival curves of these mice are shown. The survival curve for *Mx1-CreCbfb^+/56M^* mice is the same as shown in Figure 1C. Statistical significance was assessed using the log-rank test. ****P < 0.0001 compared with *Runx1^+/R188Q^Mx1-CreCbfb^+/56M^* mice. (**C**) Representative FACS plots of bone marrow cells from mice treated with pIpC for 8 weeks, gated on single cells (top plots), Lin^–^ cells (middle plots), and LK cells (bottom plots). *N* = 3–11 mice per genotype. (**D**) Dot plot showing the percentages (mean ± SEM) of populations in the marrow for each indicated genotype, as presented in **C**, with each dot representing an individual sample. Statistical significance was assessed using 2-tailed Student’s *t* test for comparing between 2 groups (AMP population) and 1-way ANOVA followed by Tukey’s post hoc test for comparisons among multiple groups (all other populations except AMP). *P < 0.05, **P < 0.01, ***P < 0.001, ****P < 0.0001, each compared with the control. ^#^*P* < 0.05, ^##^*P* < 0.01, each compared with the *Mx1-CreCbfb^+/56M^* group.

To determine the importance of RUNX1 DNA binding for leukemogenesis by CBFβ-SMMHC, we crossed a mouse strain carrying the RUNX1-R188Q mutation (*Runx1^+/R188Q^*) (39) with the *Mx1-CreCbfb^+/56M^* mice, resulting in *Runx1^+/R188Q^Mx1-CreCbfb^+/56M^* mice. Significant increase of c-Kit^+^ leukemic cells was detected in the peripheral blood of *Mx1-CreCbfb^+/56M^* mice but not in *Runx1^+/R188Q^Mx1-CreCbfb^+/56M^* or *Runx1^+/R188Q^Mx1-Cre* mice 11 weeks after pIpC treatment to induce the expression of *Cbfb-MYH11* (Figure 5A and Supplemental Figure 8A). The *Mx1-CreCbfb^+/56M^* mice also developed marked leukocytosis, severe thrombocytopenia, and progressive anemia (Supplemental Figure 8B). As expected, *Mx1-CreCbfb^+/56M^* mice died from AML with a median survival of 118.5 days, while *Runx1^+/R188Q^Mx1-CreCbfb^+/56M^* and *Runx1^+/R188Q^Mx1-Cre* mice were free of hematopoietic malignancy 52 weeks after pIpC treatment (Figure 5B and Supplemental Figure 8, C and D). These results suggest that the DNA binding ability of RUNX1 is required for *Cbfb-MYH11* to induce leukemia.

To rule out the possibility that the reason *Runx1^+/R188Q^Mx1-CreCbfb^+/56M^* mice did not develop leukemia was because of RUNX1 haploinsufficiency, we examined *Runx1^+/f^Mx1-CreCbfb^+/56M^* mice, in which one *Runx1* allele can be deleted by Cre. These mice developed leukemia with a median survival of 164 days (Supplemental Figure 9A), which is modestly prolonged in comparison with *Runx^+/+^Mx1-CreCbfb^+/56M^* mice (Figure 5B). Similar to the leukemia developed in *Mx1-CreCbfb^+/56M^* mice, increased c-Kit^+^ leukemic cells were detected in peripheral blood, spleen, and bone marrow of diseased *Runx1^f/+^Mx1-CreCbfb^+/56M^* mice (Supplemental Figure 9B). These findings indicate that RUNX1 haploinsufficiency alone does not prevent leukemogenesis, supporting a model in which RUNX1 DNA-binding activity is important for leukemogenesis.

### AMP cells from Mx1-CreCbfb^+/56M^ and Runx1^+/R188Q^Mx1-CreCbfb^+/56M^ mice exhibit distinct gene expression profiles.

The AMP population harbors pre-leukemia-initiating cells in *Mx1-CreCbfb^+/56M^* mice (25). Interestingly, we found that the AMP population was present in the *Runx1^+/R188Q^Mx1-CreCbfb^+/56M^* mice, and at a higher proportion (Figure 5, C and D). The absence of leukemia in *Runx1^+/R188Q^Mx1-CreCbfb^+/56M^* mice strongly suggests that the leukemia-initiating activity is broadly impaired across all hematopoietic populations, including the AMP population. We then hypothesized that the AMP population observed in *Runx1^+/R188Q^Mx1-CreCbfb^+/56M^* mice is functionally different from that observed in *Mx1-CreCbfb^+/56M^* mice with loss of leukemia-initiating ability, similar to that observed in *Runx1^fl/fl^Mx1-CreCbfb^+/56M^* mice (18).

**Figure 6:**
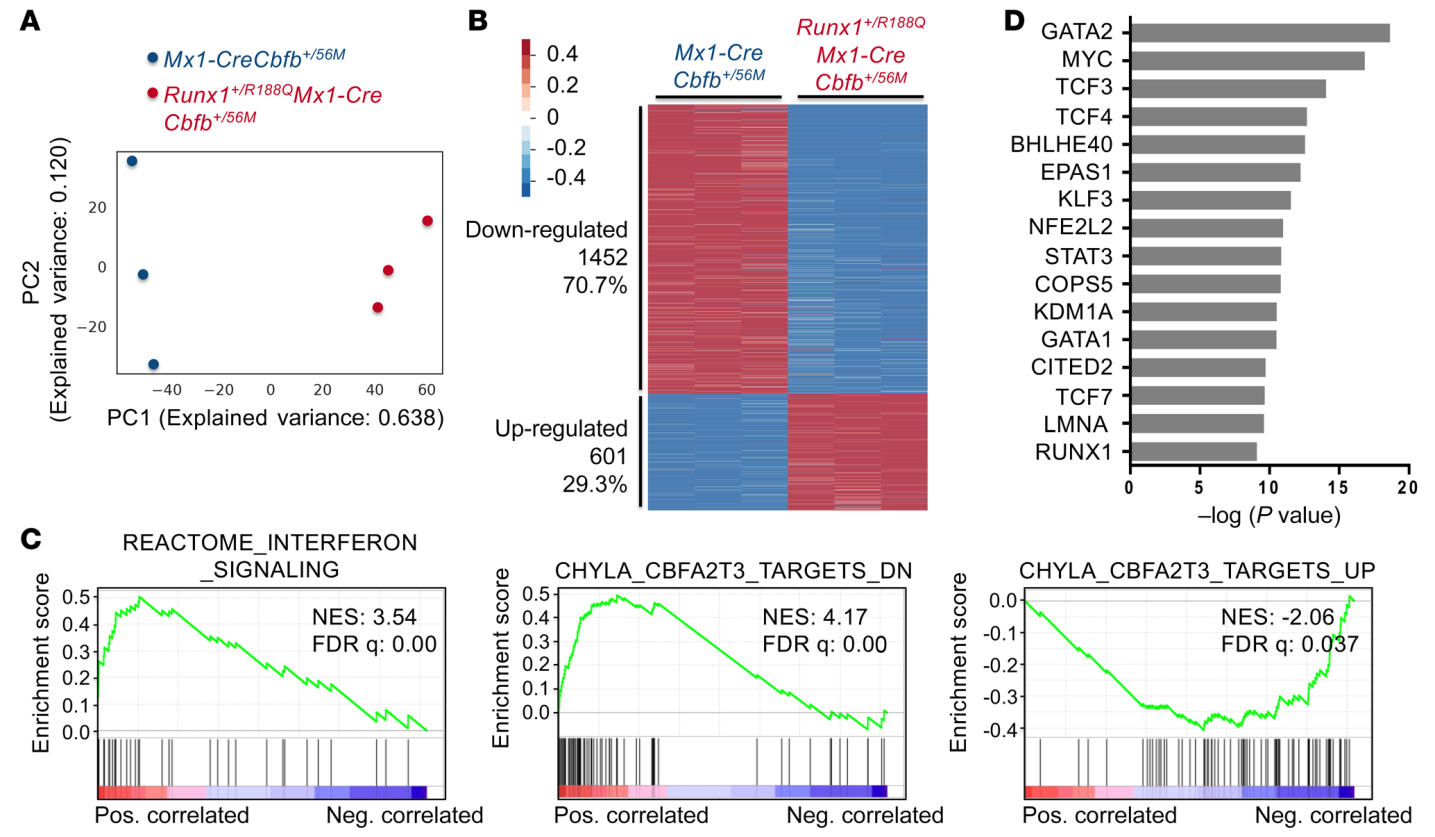
Expression of *Runx1-R188Q* alters *Cbfb-MYH11*–induced gene expression changes in the AMP population. (**A**–**D**) RNA-Seq was performed on AMP cells isolated from *Mx1-CreCbfb^+/56M^* and *Runx1^+/R188Q^Mx1-CreCbfb^+/56M^* mice 8 weeks after pIpC treatment. *N* = 3 for each genotype. (**A**) Principal component analysis plot showing clear separation between these 2 genotype groups. (**B**) Heatmap representation of identified DEGs between these 2 groups. Numbers and percentages of DEGs in each of the 2 expression clusters (down- and upregulated in *Runx1^+/R188Q^Mx1-CreCbfb^+/56M^* mice) are listed on the left. (**C**) GSEA (pre-ranked) identified curated genes sets significantly enriched in the DEGs shown in **B**. (**D**) Upstream regulators associated with DEGs shown in **B**, identified by Ingenuity Pathway Analysis.

To test this hypothesis, we first performed bulk RNA-Seq to profile gene expression changes in AMP cells isolated from *Mx1-CreCbfb^+/56M^* and *Runx1^+/R188Q^Mx1-CreCbfb^+/56M^* mice 8 weeks after pIpC injection. PCA analysis showed a clear separation of the AMP cells in *Mx1-CreCbfb^+/56M^* mice from those in the *Runx1^+/R188Q^Mx1-CreCbfb^+/56M^* mice (Figure 6A). Compared with *Mx1-CreCbfb^+/56M^* mice, 2,053 genes were differentially expressed (*q* value < 0.01, absolute fold change ≥ 2) in *Runx1^+/R188Q^Mx1-CreCbfb^+/56M^* mice, with 29.3% upregulated and 70.7% downregulated (Figure 6B and Supplemental Table 5). Interestingly, the DEGs identified in AMP cells between *Mx1-CreCbfb^+/56M^* mice and *Runx1^+/R188Q^Mx1-CreCbfb^+/56M^* mice differed from those identified in AMP cells between *Mx1-CreCbfb^+/56M^* mice and *Runx1^fl/fl^Mx1-CreCbfb^+/56M^* mice 2–3 weeks after pIpC treatment (18), with only a few overlapping genes (Supplemental Figure 10). These findings suggest that *Runx1^+/R188Q^* and *Runx1^–/–^* have distinct mechanisms preventing leukemogenesis by *Cbfb-MYH11*. Alternatively, the observed differences may reflect variations in the developmental stages of the AMP population. GSEA (35) was performed with the DEGs between AMP cells isolated from *Mx1-CreCbfb^+/56M^* and *Runx1^+/R188Q^Mx1-CreCbfb^+/56M^* mice. Several curated gene sets were significantly enriched. As shown in Figure 6C and Supplemental Table 6, the upregulated DEGs in *Runx1^+/R188Q^Mx1-CreCbfb^+/56M^* cells were positively correlated with several sets related to immune response, such as REACTOME_INTERFERON_SIGNALING, consistent with the finding that RUNX1 can negatively regulate inflammatory cytokine production (36). Interestingly, upregulated DEGs in *Runx1^+/R188Q^Mx1-CreCbfb^+/56M^* cells positively correlated with CHYLA_CBFA2T3_TARGETS_DN and negatively correlated with CHYLA_CBFA2T3_TARGETS_UP, suggesting a critical role for CBFA2T3 that may contribute to the loss of leukemia initiation ability in *Runx1^+/R188Q^Mx1-CreCbfb^+/56M^* mice. These data align with the GSEA data for DEGs between leukemogenic *Cbfb-MYH11* variants and controls, which also identified pathways related to the immune response and CBFA2T3 targets (Figure 4E), highlighting the importance of these pathways in the development of *Cbfb-MYH11*–induced leukemia. In addition, Gene Ontology analysis showed that downregulated DEGs were associated with terms such as cell division and proliferation and upregulated DEGs were associated with terms such as defense response to virus (Supplemental Figure 11).

Upstream Ingenuity Pathway Analysis revealed that, as expected, RUNX1 was one of the most significant putative upstream transcription factors regulating the DEGs in the AMP cells of *Runx1^+/R188Q^Mx1-CreCbfb^+/56M^* cells (Figure 6D). Interestingly, GATA2 and MYC were the most significant putative upstream transcription factors (Figure 6D), suggesting that RUNX1 exerts its function in part through other key hematopoietic transcription factors, such as GATA2 and MYC. These findings align with previous observations that CBFβ-SMMHC modulates the function of GATA2 for leukemogenesis (40), *Gata2* deficiency delays leukemogenesis by *Cbfb-MYH11* (41), and *Myc* is required for the survival of inv(16) leukemia-initiating cells (42). These results suggest that the AMP cells from *Mx1-CreCbfb^+/56M^* and *Runx1^+/R188Q^Mx1-CreCbfb^+/56M^* mice exhibit distinct gene expression profiles, which likely contribute to their differing leukemia-initiating capacities in mice.

### Identification of a leukemia-initiating subpopulation in the AMP cells.

**Figure 7:**
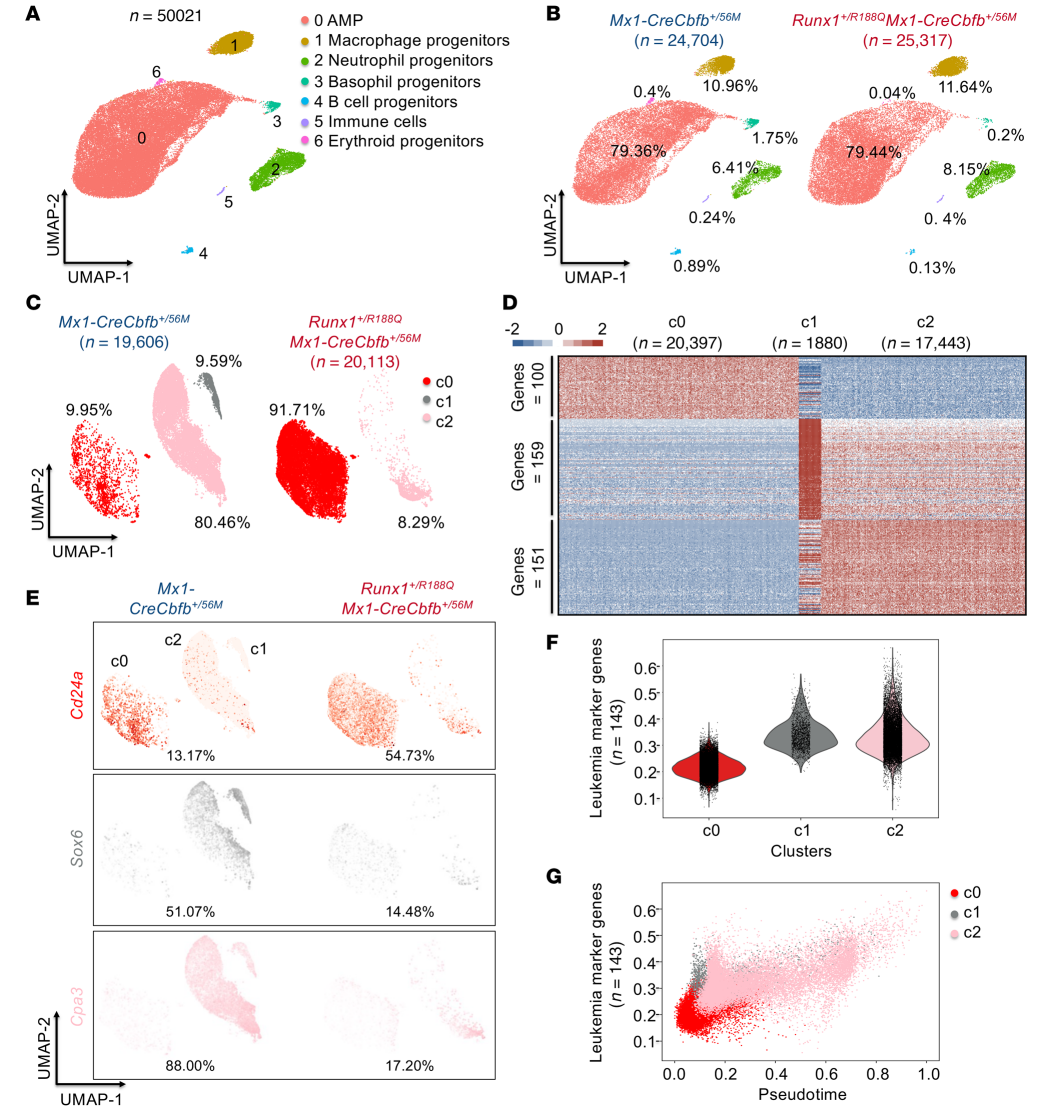
Identification of leukemia-initiating subpopulations within AMP cells by scRNA-Seq. (**A**–**F**) scRNA-Seq was performed on AMP cells isolated from *Mx1-CreCbfb^+/56M^* and *Runx1^+/R188Q^Mx1-CreCbfb^+/56M^* mice 8 weeks after pIpC treatment. (**A**) UMAP visualization of clusters identified using Seurat, including all cells from both genotypes. The total number of cells is annotated, along with the identified cell types and their respective percentages. (**B**) Detailed UMAP from **A**, with separation by genotypes and labeled percentage of cells in each cluster. (**C**) UMAP visualization of the subclusters identified in cluster 0 (AMP) in **A** for each genotype, with the percentage of cells in each subcluster indicated. (**D**) Heatmap of marker genes (*P* < 1 × 10^–10^, absolute fold change ≥2) in clusters (cells from both conditions were used) identified in **C**. Numbers of marker genes in each cluster are listed on the left. (**E**) Feature plot depicting the expression of marker genes *Cd24a*, *Sox6*, and *Cpa3* for clusters c0, c1, and c2, respectively. Colors indicate the marker gene associated with each cluster as identified in **C**, and color intensity represents expression levels in each cell. The percentages of cells expressing the indicated gene are also shown. (**F**) Violin plot illustrating the expression levels of the leukemia genes across all clusters in **C**. (**G**) Pseudotime graph depicting the leukemia progression of the specified clusters in **C**.

We performed single-cell RNA-Seq (scRNA-Seq) on AMP cells to investigate their heterogeneity and identify subpopulations affected by RUNX1-R188Q. More than 50,000 AMP cells were collected from 4 mice, 2 from *Mx1-CreCbfb^+/56M^* and 2 from *Runx1^+/R188Q^Mx1-CreCbfb^+/56M^* (Supplemental Figure 12A), and these 2 biological replicates exhibited high consistency shown by the single-cell aggregated expression profiles (Supplemental Figure 12B). We therefore pooled cells with the same genotype from the 2 replicates for further analysis. After integration of the combined dataset in Seurat (43), a total of 7 clusters were identified (Figure 7A), similar to the clusters observed in the previous analysis (44). The cellular identity of each cluster in Figure 7A was determined based on the detected marker genes specific to each cluster (Supplemental Table 7) and the expression of known hematopoietic lineage markers (44–46). As expected, most of the cells (79.36% for *Mx1-CreCbfb^+/56M^* mice and 79.44% for *Runx1^+/R188Q^Mx1-CreCbfb^+/56M^* mice) were in the AMP population (Figure 7B). To explore the heterogeneity within the AMP population, we selected cells from cluster 0 for further reclustering analysis with a method similar to that in our previous study (18), which identified a total of 3 clusters (Figure 7C). Interestingly, there were major shifts in the proportion of the clusters between *Mx1-CreCbfb^+/56M^* and *Runx1^+/R188Q^Mx1-CreCbfb^+/56M^* mice (Figure 7C). In *Mx1-CreCbfb^+/56M^* mice, the most abundant cluster was c2 (80.46%), whereas in *Runx1^+/R188Q^Mx1-CreCbfb^+/56M^* mice, the most abundant cluster was c0 (91.71%). Notably, cluster c1 (9.59%) was only present in the *Mx1-CreCbfb^+/56M^* mice. To explore the leukemogenic role of these clusters, we conducted an integrated analysis to identify marker genes across clusters c0, c1, and c2, followed by Gene Ontology term enrichment analysis. A total of 100 (e.g., *Cd24a*), 159 (e.g., *Sox6*), and 151 (e.g., *Cpa3*) marker genes were identified in clusters c0, c1, and c2 respectively (Figure 7, D and E, and Supplemental Table 8). Gene Ontology analysis revealed that cluster c0 marker genes were enriched for terms related to inflammatory response, cluster c1 marker genes were associated with terms related to myeloid cell development, and cluster c2 marker genes were linked to cell-cell adhesion and leukocyte differentiation (Supplemental Figure 12C), suggesting that each cluster represents a distinct functional state.

In our previous scRNA-Seq study on pre-leukemia and leukemia cells in *Mx1-CreCbfb^+/56M^* mice, we identified clusters enriched in leukemic mice (clusters B and J in Figure 1E of ref. 45) (44). We first extracted the top 100 marker genes from those 2 clusters (B and J), resulting in a total of 143 marker genes due to overlapping genes between the clusters (Supplemental Table 9). We then determined the expression levels of these 143 leukemia marker genes in the 3 AMP subclusters (Figure 7C), and found that they were more highly expressed in clusters c1 and c2 than in cluster c0 (Figure 7F). We then performed a trajectory analysis of the 3 clusters with the PAGA algorithm in the Scanpy package (47) to generate a pseudotime graph, with cluster c2 as the root cluster. Next, we reversed the pseudotime to model the leukemic cell development trajectory from non-leukemia cells to leukemia cells (Figure 7G). This analysis revealed that cluster c0, enriched in AMP cells from *Runx1^+/R188Q^Mx1-CreCbfb^+/56M^* mice, represents an early stage of leukemia development. In contrast, clusters c1 and c2, which are enriched in AMP cells from *Mx1-CreCbfb^+/56M^* mice, correspond to later stages. Specifically, cluster c1 represents an intermediate stage, while cluster c3 reflects a more advanced leukemic state (Figure 7G). Collectively, these results suggest that the AMP cells in *Runx1^+/R188Q^Mx1-CreCbfb^+/56M^* mice had lower leukemia potential, further confirming that DNA binding by RUNX1 is required for the generation and maintenance of functional AMP population for leukemia.

### Cytoplasmic sequestration of RUNX1 by CBFβ-SMMHC is insufficient to drive leukemogenesis.

**Figure 8:**
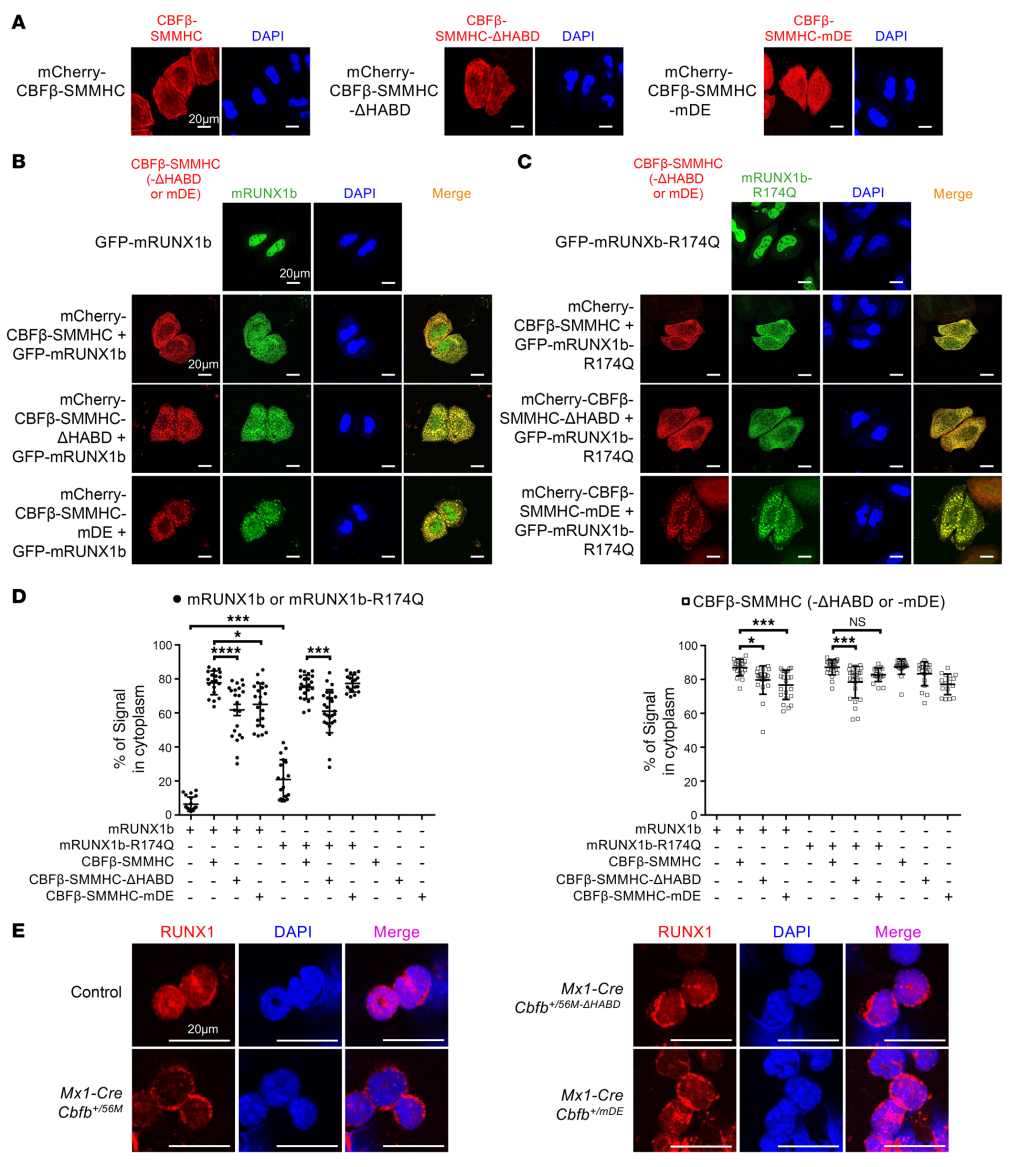
Cytoplasmic sequestration of RUNX1 by CBFβ-SMMHC is dispensable for leukemia initiation. (**A**–**D**) HeLa cells were transfected with the indicated plasmids, and confocal microscopy was used to determine the localization of the specified proteins. Images shown are representative of 2–3 independent experiments. (**A**) Representative images of cells transfected with mCherry-tagged CBFβ-SMMHC or related mutants alone. (**B**) Representative images of cells transfected with GFP-tagged mRUNX1b alone (top panels) or cotransfected with mCherry-CBFβ-SMMHC or related mutants (bottom panels). (**C**) Representative images of cells transfected with GFP-tagged mRUNX1b-R174Q alone (top panels) or cotransfected with mCherry-CBFβ-SMMHC or related mutants (bottom panels). (**D**) Zeiss Arivis software was used to quantify the signals of the indicated protein in the cytoplasm and nucleus of cells transfected with the specified plasmids. The percentage of the protein signal in the cytoplasm is shown (mean ± SEM), with each dot representing an individual cell. Statistical significance was assessed using 1-way ANOVA followed by Tukey’s post hoc test. *P < 0.05, ***P < 0.001, ****P < 0.0001. (**E**) Representative images of bone marrow cells from mice 2–3 weeks after pIpC treatment, stained for RUNX1 to assess its localization. Scale bars: 20 μm for all panels. Images shown are representative of 2 independent experiments.

It has been shown by many groups, including ours, that CBFβ-SMMHC sequesters most RUNX1 to the cytoplasm, supporting a dominant-negative model for CBFβ-SMMHC–induced leukemia development (8, 19, 48, 49). To investigate the significance of cytoplasmic sequestration of RUNX1 by CBFβ-SMMHC for leukemogenesis, we performed immunofluorescence assay by transfecting HeLa cells with mRUNX1b and/or CBFβ-SMMHC or related mutants. We found that the transfected CBFβ-SMMHC and the two CBFβ-SMMHC mutants mainly located in the cytoplasm (Figure 8A). However, filament-like structures were only observed in cells transfected with CBFβ-SMMHC and CBFβ-SMMHC-ΔHABD, but not in those transfected with CBFβ-SMMHC-mDE (Figure 8A), consistent with the filament formation ability of purified proteins observed in vitro (Figure 2D). Interestingly, both mRUNX1b and its mutant mRUNX1b-R174Q were predominantly found in the nuclei when transfected alone (Figure 8, B and C). When mRUNX1b was cotransfected with CBFβ-SMMHC or the two CBFβ-SMMHC mutants, mRUNX1b was sequestered to the cytoplasm, forming aggregate-like structures together with the CBFβ-SMMHC variants (Figure 8, B and D). These aggregate-like structures, instead of the filament-like structures shown in Figure 2D, are possibly due to interactions with other proteins within the cells. Similar results were observed in cells cotransfected with mRUNX1b-R174Q and CBFβ-SMMHC or the two CBFβ-SMMHC mutants, which is known to block leukemogenesis in *Cbfb-MYH11*–expressing mice (*Runx1^+/R188Q^Mx1-CreCbfb^+/56M^*; Figure 8, C and D).

To assess whether the cytoplasmic sequestration of RUNX1 by CBFβ-SMMHC and its mutants also occurs in hematopoietic cells, we performed colocalization experiments using bone marrow cells isolated from *Mx1-CreCbfb^+/56M^*, *Mx1-CreCbfb^+/56M-ΔHABD^*, and *Mx1-CreCbfb^+/mDE^* mice. Because of poor antibody performance for SMMHC detection in these cells, we focused on RUNX1 localization. Immunofluorescence staining revealed that, similar to the phenotype observed in HeLa cells, RUNX1 was mislocalized to cytoplasmic aggregates in bone marrow cells expressing full-length CBFβ-SMMHC, CBFβ-SMMHC-ΔHABD, or CBFβ-SMMHC-mDE, but not in control bone marrow cells (Figure 8E). These findings confirm that the sequestration phenotype is not cell type specific and is observed in both non-hematopoietic and hematopoietic cells. Therefore, our data suggest that cytoplasmic sequestration of RUNX1 by CBFβ-SMMHC is insufficient by itself to drive leukemogenesis, while our findings do not fully exclude the possibility that this process contributes to leukemogenesis in the context of CBFβ-SMMHC. Further investigation will be necessary to determine whether cytoplasmic sequestration is dispensable for fusion-driven leukemogenesis.

## Discussion

Our recent data suggested that *Runx1* is indispensable for *Cbfb-MYH11*–induced leukemogenesis, functioning cooperatively with CBFβ-SMMHC to regulate critical genes for leukemia initiation (18). Along with other recent findings (22, 24, 50–53), these data challenged the RUNX1-repression model and underscore the essential role of RUNX1 in *CBFB-MYH11*–driven leukemia development. However, the underlying mechanism remains unclear. In this study, we conducted a comprehensive study on how RUNX1 contributes to leukemogenesis by *Cbfb-MYH11*. We found that leukemogenic CBFβ-SMMHC variants, including full-length CBFβ-SMMHC and CBFβ-SMMHC-ΔHABD, could enhance the binding affinity of RUNX1 to its target DNA. This was demonstrated both in vitro using purified proteins via BLI assay (Figure 2A) and in vivo using ChIC-Seq assay with LK cells isolated from mice (Figure 3A). On the other hand, the level of binding affinity between RUNX1 and CBFβ-SMMHC did not correlate with leukemogenicity. To further demonstrate the importance of RUNX1-DNA binding for leukemogenesis, we generated mice with one *Runx1* allele carrying the R188Q substitution, which renders RUNX1 defective in DNA binding but still able to interact with CBFβ-SMMHC, along with *Cbfb-MYH11* (*Runx1^+/R188Q^Mx1-CreCbfb^+/56M^*). Remarkably, these mice remained free of hematopoietic malignancy 52 weeks after pIpC treatment. All together, these results suggest that enhancing RUNX1’s DNA binding affinity is a critical step for CBFβ-SMMHC-induced leukemia.

To investigate the impact of RUNX1-R188Q on *Cbfb-MYH11*–induced leukemia, we examined the AMP population in the bone marrow, as this cell population plays a critical role in *Cbfb-MYH11*–induced leukemia (25, 54). We found that the percentage of AMP population in *Runx1^+/R188Q^Mx1-CreCbfb^+/56M^* mice was higher than that in *Mx1-CreCbfb^+/56M^* mice (Figure 5, C and D). This suggests that *Cbfb-MYH11*–expressing cells with one allele of RUNX1-R188Q are still able to generate the pre-leukemic AMPs but fail to progress to full-blown leukemia. We then performed bulk RNA-Seq on the AMPs, and the results showed a large number of DEGs between *Mx1-CreCbfb^+/56M^* and *Runx1^+/R188Q^Mx1-CreCbfb^+/56M^* mice. GSEA analysis of the DEGs revealed that genes associated with immune response and CBFA2T3 knockout in immature bone marrow progenitor cells were significantly altered, emphasizing the importance of these pathways in maintaining functional AMP population (Figure 6).

In the analysis of AMP cells from *Mx1-CreCbfb^+/56M^* and *Runx1^+/R188Q^Mx1-CreCbfb^+/56M^* mice by scRNA-Seq, we revealed substantial shifts in the clusters (Figure 7C). These findings suggest that, even though the AMP population is formed, there are substantial changes at the subpopulation level, which may affect the function and leukemogenic potential of the AMP cells. We did observe major differences in DEGs and cellular heterogeneity in AMPs between this study and our previous study (comparing *Mx1-CreCbfb^+/56M^* and *Runx1^fl/fl^Mx1-CreCbfb^+/56M^* mice) (18), which may suggest a different impact of the *Runx1-R188Q* mutation compared with *Runx1* knockout. However, the cells were harvested at 8 weeks after pIpC treatment in this study, while the cells were harvested 2–3 weeks after pIpC treatment in our previous study. Therefore, the observed variations may reflect differences in AMP developmental stages rather than distinct underlying molecular mechanisms. Further studies are needed to elucidate whether these functional disparities arise from unique molecular mechanisms or stage-specific influences on leukemogenesis. Importantly, we were able to demonstrate that the clusters (c1 and c2) with the highest expression levels of leukemia-related genes were markedly reduced in the *Runx1^+/R188Q^Mx1-CreCbfb^+/56M^* mice, with a corresponding increase of cluster c0, which appeared to be less advanced in leukemogenesis in the pseudotime trajectory plot and to have lower expression levels of the leukemia genes.

We also conducted bulk RNA-Seq on LK cells isolated from mice expressing *Cbfb-MYH11* or its related mutant to identify genes involved in *Cbfb-MYH11*–induced leukemia development. The analysis revealed that most DEGs were upregulated in both *Mx1-CreCbfb^+/56M^* and *Mx1-CreCbfb^+/56M-ΔHABD^* mice. Genes associated with inflammatory responses and CBFA2T3 knockout were significantly enriched among these DEGs. Notably, these pathways were also enriched in the DEGs identified in the AMP population between *Mx1-CreCbfb^+/56M^* and *Runx1^+/R188Q^Mx1-CreCbfb^+/56M^* mice, further emphasizing their critical role in *Cbfb-MYH11*–induced leukemia. Interestingly, increased enrichment of RUNX1 and CBFβ-SMMHC binding at TSSs for DEGs in all clusters was observed in mice expressing leukemogenic CBFβ-SMMHC variants, compared with those expressing the non-leukemogenic CBFβ-SMMHC-mDE. These results suggest that RUNX1 and CBFβ-SMMHC frequently function together as a transcriptional activator complex, binding to target genes and regulating their expression to drive leukemogenesis. This aligns with our previous findings (18), highlighting the transactivation role of CBFβ-SMMHC in the development of leukemia.

Interestingly, GSEA analysis of DEGs from RNA-Seq data between mice expressing leukemogenic CBFβ-SMMHC proteins and those expressing non-leukemogenic CBFβ-SMMHC proteins identified CBFA2T3 target genes. Specifically, upregulated genes in immature bone marrow progenitors upon knockout of CBFA2T3 were enriched in bone marrow cells from mice expressing leukemogenic CBFβ-SMMHC proteins. Conversely, downregulated genes in immature bone marrow progenitors upon knockout of CBFA2T3 were enriched in bone marrow cells from *Runx1^+/R188Q^Mx1-CreCbfb^+/56M^* mice that did not develop leukemia. *CBFA2T3*, also known as *RUNX1T3*, *MTG16*, and *ETO2*, encodes a transcription corepressor and has been linked to maintenance of hematopoietic stem cell stemness, expansion of leukemia stem cells, AML relapse, and inhibition of myeloid differentiation. CBFA2T3 is frequently targeted by the t(16;21) translocation in leukemia, resulting in a *RUNX1-RUNX1T3* fusion gene, which mimics the function of *RUNX1-RUNX1T1* for leukemogenesis (55). Our findings here may suggest a link between *CBFB-MYH11* leukemia and *RUNX1-RUNX1T1* leukemia.

CBFβ-SMMHC-ΔHABD reduces RUNX1 chromatin binding, but still leaves it much higher than CBFβ-SMMHC-mDE (Figure 3A), suggesting that partial preservation of RUNX1 binding may contribute to its leukemogenic activity. The lower RUNX1 chromatin binding seems to correlate with longer latency for leukemia development in the *Mx1-CreCbfb^+/56M-ΔHABD^* mice. However, the leukemogenic ability of CBFβ-SMMHC-ΔHABD may not be explained solely by partial preservation of RUNX1 DNA binding. Notably, CBFβ-SMMHC-ΔHABD induced a distinct transcriptional program (Figure 4B, clusters c3 and c5), raising the possibility that deletion of the HABD alters interactions with RUNX1-associated cofactors or other transcriptional regulators. These findings support a model in which the CBFβ-SMMHC-ΔHABD-ΔHABD mutant reprograms transcription through mechanisms beyond RUNX1 sequestration or chromatin binding, potentially by modifying cofactor recruitment dynamics, a hypothesis that warrants further investigation in future studies.

As expected, immunofluorescence assays revealed that in cultured HeLa cells cotransfected with mRUNX1b and CBFβ-SMMHC, CBFβ-SMMHC sequestered most mRUNX1b to the cytoplasm (Figure 8, B and D), consistent with a dominant-negative model for CBFβ-SMMHC function (8, 19, 48, 49). However, cytoplasmic sequestration of mRUNX1b was also observed in cells cotransfected with mRUNX1b and leukemogenic CBFβ-SMMHC-ΔHABD, as well as in cells cotransfected with mRUNX1b and non-leukemogenic CBFβ-SMMHC-mDE (Figure 8, B and D). Moreover, similar results were observed in cells cotransfected with mRUNX1b-R174Q and these fusion proteins (Figure 8, C and D), despite the absence of leukemia development in *Runx1^+/R188Q^Mx1-CreCbfb^+/56M^* mice. These findings highlight that the cytoplasmic sequestration of RUNX1 or RUNX1-R174Q by these fusion proteins does not fully explain their distinct leukemogenic potentials, suggesting that additional mechanisms beyond RUNX1 cytoplasmic sequestration are involved in CBFβ-SMMHC–mediated leukemogenesis. In conclusion, our findings reported in this study support a model of direct transcriptional dysregulation by RUNX1/CBFβ-SMMHC that induces proinflammatory and signal transduction pathways, underscoring the pivotal role of altered transcriptional regulation in leukemogenesis.

Currently, the standard treatment for inv(16) AML is nonselective cytotoxic chemotherapy, which often yields a strong initial response (56). However, approximately 50% of patients eventually experience relapse (57). Given the pivotal role of CBFβ-SMMHC in leukemogenesis, several efforts have been made to develop targeted therapies to inhibit its activity, mainly through small-molecular inhibitors that disrupt the CBFβ-SMMHC:RUNX1 interaction (42, 58, 59). Our findings suggest that small molecules targeting RUNX1-DNA interaction may offer a more effective and less toxic therapeutic option for patients with inv(16) AML.

## Methods

### Sex as a biological variable.

Our study examined male and female animals, and similar findings are reported for both sexes.

### Animals.

*Cbfb-MYH11* conditional knockin (*Cbfb^+/56M^*) mice (25), *Mx1*-*Cre* mice (26), and *Runx1* heterozygous conditional knockout (*Runx1^+/f^*) mice (60) have been described previously and were backcrossed for more than 10 generations onto the C57BL/6J strain. The mouse strain carrying the RUNX1-R188Q mutation (corresponding to R201Q mutation in human RUNX1c; *Runx1^+/R188Q^*) was provided in-house (39) and was backcrossed over 10 generations onto the C57BL/6N strain. All these mice were genotyped by PCR with gene-specific primers (Supplemental Table 1) using tail-snip DNA prepared with a DNeasy Blood & Tissue Kit (QIAGEN). C57BL/6-CD45.1 (B6.SJL-PtprcaPepCb/BoyJ) and 129/SvEv (129S6/SvEvTac) mice were purchased from The Jackson Laboratory. Eight- to 24-week-old mice and their littermate controls were injected intraperitoneally with 250 μg of pIpC (InvivoGen) every other day for 3 doses to induce the expression of *Cbfb-MYH11* or *Cbfb-MYH11*-ΔHABD. Mice were then observed for leukemia development over the course of 52 weeks after pIpC injections.

### Plasmids.

*Runx1b* cDNA sequence was cloned into pEGFP-C1 (Addgene). *CBFB-MYH11*, *CBFB-MYH11*-ΔHABD, and *CBFB-MYH11*-mDE cDNA sequences were cloned into mCherry-C1 vector (Addgene), respectively. *RUNX1c*-R201Q–related plasmids were generated by QuikChange II XL Site-Directed Mutagenesis Kit (Agilent) based on the related *RUNX1* plasmids (PCDNA3.1). The RHD domain of hRUNX1c and CBFB was cloned into pET28a vector (SnapGene) and *CBFB-MYH11*, *CBFB-MYH11*-ΔHABD, and *CBFB-MYH11*-mDE were cloned into pET30a vector (SnapGene) to express indicated proteins in *E*. *coli*.

### Flow cytometry.

Peripheral blood, spleen, and bone marrow cells from mice were isolated and stained as described previously (17). Lineage cells were determined by staining with lineage-specific antibodies including CD3-PE (12-0031-83), CD4-PE (12-0042-83), CD8-PE (12-0081-83), B220-PE (12-0452-83), Gr1-PE (12-5931-83), Mac1-PE (12-0112-83), and Ter119-PE (12-5921-83). Other fluorescence-conjugated anti-mouse antibodies used in this study include CD19-PE (12-0199-42), CD3-PerCP-cyanine5.5 (Cy5.5) (45-0031-82), CD4-PerCP-Cy5.5 (45-0042-82), CD8-PerCP-Cy5.5 (45-0081-82), Sca1–Pacific blue (108120), Mac1-APC-eFluor 780 (47-0112-82), c-Kit–APC (17-1171-83), CD34-FITC (11-0341-85), and FcγRII/III-PE-Cy7 (25-0161-82). All antibodies were purchased from Thermo Fisher Scientific, except for Sca1–Pacific blue, which was obtained from BioLegend. Data were acquired using a CytoFLEX S Flow Cytometer (Beckman Coulter) and analyzed using FlowJo software (BD Biosciences).

### Western blot analysis.

Western blot was performed with standard protocols (Invitrogen, NuPAGE electrophoresis system). Internal control GAPDH was detected with anti-GAPDH (Abcam, ab8245), followed by anti-mouse IgG-HRP antibody (Vector Laboratories, PI-2000-1). RUNX1 and CBFβ/CBFβ-SMMHC were detected with anti-RUNX1 (Abcam, ab92336) and anti-CBFβ (Abcam, ab33516) antibodies, respectively, followed by anti-rabbit IgG-HRP antibody (Vector Laboratories, PI-1000). Lamin B was detected with anti–lamin B1 (HRP) antibody (Abcam, ab194109).

### BLI assay.

His-tagged RHD, CBFβ, CBFβ-SMMHC, CBFβ-SMMHC-ΔHABD, and CBFβ-SMMHC-mDE were expressed in *E*. *coli* BL21 (DE3, Thermo Fisher Scientific) and purified using Ni-NTA Agarose affinity chromatography (QIAGEN). BLI assay was conducted using standard protocols on the Octet RED96e system (ForteBio, Sartorius). Briefly, to detect interactions between RHD and CBFβ, CBFβ-SMMHC, or their mutants, purified RHD was biotinylated using EZ-Link NHS-PEG4-Biotin (Thermo Fisher Scientific) and purified with a protein desalting spin column (Thermo Fisher Scientific) according to the manufacturer’s instructions. The BLI assay was performed as follows. Step 1 (baseline): Streptavidin biosensor tips were placed in buffer 1 (20 mM Tris-HCl [pH 8.0], 100 mM KCl, 1 mM EDTA, 1 mM DTT, and 0.02% Tween 20) for an initial baseline reading. Step 2 (loading): Biotinylated RHD in buffer 1 was loaded onto the sensors. Step 3 (baseline): Sensors were returned to buffer 1 for another baseline reading. Step 4 (association): Sensors were dipped into wells containing CBFβ, CBFβ-SMMHC, or related mutants at varying concentrations in buffer 1 to allow binding. Step 5 (dissociation): Sensors were then dipped back into buffer 1 to monitor dissociation kinetics. To detect interactions between target DNA oligonucleotides and RHD or its protein complexes, biotin-labeled DNA oligonucleotides were directly synthesized by Integrated DNA Technologies. The BLI assay was conducted as follows. Step 1 (baseline): Streptavidin biosensor tips were placed in buffer 2 (20 mM Tris-HCl [pH 8.0], 150 mM KCl, 1 mM EDTA, 1 mM DTT, 0.02% Tween-20, and 800 nM CBFβ, 400 nM CBFβ-MYH11, or related mutants) to establish an initial baseline reading. Step 2 (loading): Biotinylated DNA oligonucleotides in buffer 2 were immobilized onto the streptavidin biosensor tips. Step 3 (baseline): Sensors were returned to buffer 2 for a secondary baseline reading. Step 4 (association): Sensors were dipped into wells containing RHD preincubated at 37°C for 30 minutes in buffer 2 to allow the binding of RHD with CBFβ, CBFβ-SMMHC, or related mutants. Step 5 (dissociation): Sensors were returned to buffer 2 to monitor dissociation kinetics.

### Negative staining–electron microscopy.

Negative staining was performed using a standard protocol as previously described (61). Briefly, 5 μL of purified protein (0.2 mg/mL) or protein complex (0.2 mg/mL of each), preincubated at 4°C for 1 hour in a buffer containing 20 mM Tris-HCl (pH 8.0), 50 mM KCl, 1 mM EDTA, and 1 mM DTT, was applied to a glow-discharged, carbon-coated 400-mesh copper grid for 30 seconds. The grid was then stained with a 2% (wt/vol) uranyl formate solution (pH 4.6) for 10 seconds, with the staining repeated 2 additional times. After air-drying, the grid was ready for transmission electron microscopy analysis.

### Luciferase reporter assay.

293T cells (American Type Culture Collection [ATCC]) were cultured in DMEM supplemented with 10% FBS following ATCC recommendations. The cells were transfected with a CSF1R promoter–driven luciferase reporter plasmid (*CSF1R*-reporter) (38), a control reporter plasmid (PRL-TK), and plasmids encoding the indicated proteins. After 48 hours, luciferase activity was measured using the Dual-Luciferase Reporter Assay System (Promega), following the manufacturer’s instructions.

### EMSA.

EMSA was performed following the IRDye oligonucleotide protocol from LICOR. Briefly, RUNX1 or RUNX1c-Q201Q proteins were generated using the TnT Quick Coupled Transcription/Translation System (Promega), according to the manufacturer’s instructions. A 20-nucleotide sequence from the CSF1R promoter (5′-CAAACTCTGTGGTTGCCTTG-3′) labeled with IRDye700 (Integrated DNA Technologies) was used as the DNA probe. Proteins were incubated with the probe at room temperature for 30 minutes, followed by electrophoresis in 4%–12% TBE gels with 1× TBE. Gels were then imaged using an Odyssey Imager (LICOR).

### Immunofluorescence.

HeLa cells (ATCC) were cultured in DMEM containing 10% FBS according to the recommendations of ATCC. HeLa cells grown on cover glasses were transfected with indicated plasmids. Twenty-four hours later, the cells were fixed with 4% paraformaldehyde (PFA) for 15 minutes. Immunofluorescence staining was performed with standard protocols.

Mouse bone marrow cells were attached to glass microscopic slides with Cytospin (Shandon). The cells on the slides were then fixed with 4% PFA for 15 minutes, followed by 0.25% Triton X-100 for 10 minutes. Immunofluorescent staining was performed with standard protocols. RUNX1 protein was first stained with anti-RUNX1 antibody (Santa Cruz Biotechnology, sc-365644), followed by anti-mouse Alexa Fluor 568 fluorescent antibody (Thermo Fisher Scientific, A-11031).

Images were captured with a laser scanning confocal microscope (Zeiss) and analyzed with Zen software (Zeiss). The signal of the indicated protein in the cytoplasm and nucleus was quantified by Arivis software (Zeiss).

### RNA sequencing.

Mice of the indicated genotypes were treated with pIpC. Two to three weeks after the final pIpC injection, LK cells were isolated from bone marrow using a BD FACSAria IIIu cell sorter (BD Biosciences). Eight weeks after the final pIpC injection, AMP cells were similarly isolated from bone marrow using the BD FACSAria IIIu cell sorter (BD Biosciences).

Total RNA from LK and AMP cells was extracted with AllPrep DNA/RNA/Protein Mini Kit (QIAGEN). Poly(A)-selected stranded mRNA libraries were constructed using Illumina TruSeq Stranded mRNA Sample Prep Kits according to the manufacturer’s instructions. Unique barcode adapters were applied to each library. All libraries were combined in equimolar proportion into one pool for sequencing with a NovaSeq 6000 sequencer. Raw reads were mapped to mouse genome mm10 with STAR (v2.5.1b) (62). The gene expression levels were quantified and normalized by cuffnorm from the cufflinks (v2.2.1) (63) package into a fragments per kilobase of transcript per million mapped reads (FPKM) matrix. Genes were filtered off if their expression levels were less than 1 FPKM in all samples. The expression matrix was further log_2_-transformed. The differentially expressed genes (DEGs) were called with cuffdiff (v2.2.1) (64), requiring *q* value <0.01, absolute fold change between the two conditions ≥2, the sum of mean expression level between the two conditions ≥1, and genes in the filtered expression matrix. The enriched Gene Ontology terms were identified by findGO.pl from the HOMER package (v4.10.4) (65), requiring at least 5 genes in the target biological process term, fewer than 500 total genes in the term, and a *P* value less than 1 × 10^–5^. Ingenuity Pathway Analysis (QIAGEN) was performed to identify upstream regulators of the DEGs.

### ChIC sequencing.

LK cells were fixed with 1% PFA and subjected to ChIC-Seq as previously described (30). Specifically, antibody/pA-MNase complex with 50,000 cells was used for each sample. RUNX1 (Abcam, ab23980), MYH11 (Diagenode, C15310254), and H3K27ac (Abcam, ab4729) antibodies were used to identify the binding sites of the indicated proteins. Rabbit IgG (Abcam, ab172730) was used as an isotype control. All libraries were combined in equimolar proportion into one pool for sequencing with a HiSeq 3000 sequencer (Illumina). Paired-end raw reads were mapped to mouse genome mm10 by Bowtie2 (v2.3.5) (66) and converted into bigwig files with reads per million (RPM) signals for aggregation analysis and visualization in WashU Epigenome Browser (67).

### Single-cell RNA sequencing.

10x Genomics chromium platform (68) was used to capture isolated AMP cells, and all steps were performed according to the manufacturer’s instructions. The Chromium Single Cell 3′ Library & Gel Bead Kit v3 was used. Libraries were sequenced on an Illumina NextSeq 550 sequencer. Reads were pre-processed with cellranger count (v7.2.0) (10x Genomics). Cells were retained for subsequent analysis if they expressed at least 500 genes, had fewer than 5% of reads mapping to mitochondrial genes, and had more than 5% of reads mapping to ribosomal genes. These cells were then normalized and integrated using Seurat (v4.2.1) (43). Integrated UMAP visualizations of cells and marker genes for the 7 populations were also obtained with Seurat. Cells classified as the AMP populations were extracted, and their normalized expression matrix was used to obtain the first 2 component values for each cell with the PCA implemented in the scikit-learn package (69). The first 2 components were then fed to UMAP (70) with key parameters: *n* = 100, init = “pca,” metric = “euclidean,” min_dist = 0.0, n_epochs = 1,000, random_state = 123. The AMP UMAP results were clustered using HDBSCAN (v0.8.33) (71) with key parameters: min_samples = 1,000, min_cluster_size = 100. Marker genes within the AMP cells among clusters (cells from both conditions were used) were identified using a 2-sided Wilcoxon’s rank-sum test implemented in the Scipy package (v1.11.3) (scipy.stats.ranksums) (72), with criteria of *P* < 1 × 10^–10^, absolute fold change ≥2, and expression in more than 20% of cells in one cluster. We reduced the gene-by-cell matrix to 600 columns by averaging groups of columns, enhancing computational efficiency and clarity for heatmap visualization.

### Statistics.

Data were analyzed using GraphPad Prism. Results are expressed as mean ± SEM. Data from multiple groups were analyzed using 1-way ANOVA followed by Tukey’s post hoc test where appropriate. Comparisons between 2 groups were performed using a 2-tailed unpaired Student’s *t* test. The differences in survival times of mice were analyzed with the Kaplan-Meier method and log-rank test. *P* less than 0.05 was considered statistically significant.

### Study approval.

Animal care and use were approved by the National Human Genome Research Institute Animal Care and Use Committee, and all the performed procedures followed relevant NIH guidelines and regulations.

### Data availability.

RNA-Seq, ChIC-Seq, and scRNA-Seq data were deposited in the NCBI’s Gene Expression Omnibus (GEO) database with accession number GSE285824. Values for all data points in figures are reported in the Supporting Data Values file. This paper does not report original code. Data analysis was conducted using published software packages as detailed and referenced in Methods.

## Author contributions

TZ designed and performed experiments, analyzed data, and wrote the paper. Y Cao analyzed the bulk RNA-Seq, ChIC-Seq, and scRNA-Seq data and wrote the paper. TD, Y Chen, GL, and ACM performed experiments. XW and JAH assisted with the immunofluorescence and data analysis. JC and LG generated the conditional *Cbfb-MYH11-ΔHABD* knockin mouse model. SA and MK assisted with flow cytometry and sorting. SW assisted with microscopy for image acquisition. BS and AGE assisted with RNA-Seq and scRNA-Seq. DW assisted with BLI experiments. LHC provided the *Runx1*-R188Q mutation mouse model. WY assisted with protein purification from *E*. *coli*. JJ assisted with in vitro experiments using purified proteins. KZ contributed to the sequencing data analysis. PPL designed the experiments, analyzed data, wrote the paper, and supervised the study. The order of the shared first authors (T Zhen and Y Cao) was determined based on relative contributions to study design, experimental work, and preparation of manuscript draft and figures.

## Supplementary Material

Supplemental data

Unedited blot and gel images

Supplemental tables 1-9

Supporting data values

## Version 1 08/05/2025

In-Press Preview

## Version 2 10/01/2025

Electronic publication
