# Supplementary material for: CBFβ-SMMHC–driven leukemogenesis requires enhanced RUNX1-DNA binding affinity in mice
Source: J Clin Invest. 2025 Aug 5;135(19):e192923. doi: 10.1172/JCI192923 (PMC12483565; doi:10.1172/JCI192923)
Supplement: Supplemental data [file jci-135-192923-s110.pdf]

Supplemental Figure 1

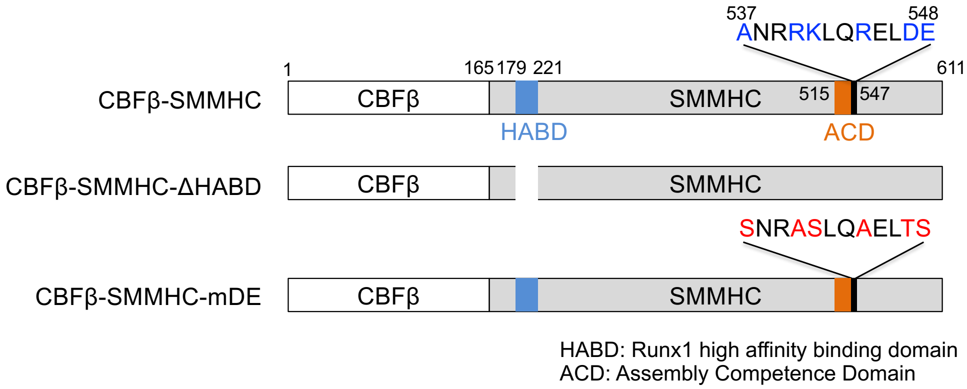

Supplemental Figure 1: Diagram illustrating the proteins encoded by *Cbfb-MYH11, Cbfb-MYH11-ΔHABD* and *Cbfb-MYH11-mDE,* with key functional domains highlighted.

Supplemental Figure 2

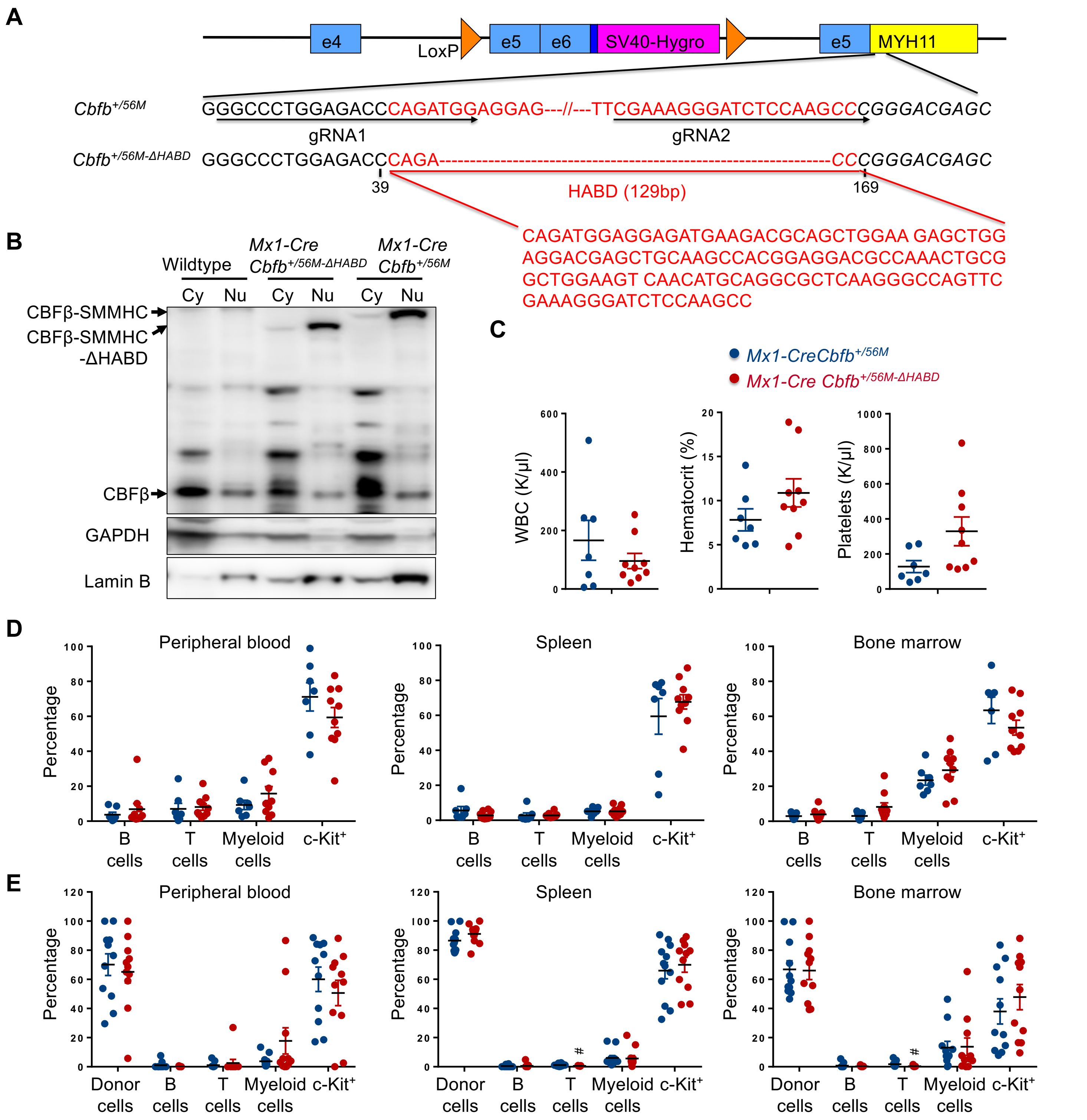

Supplemental Figure 2: Generation of conditional *Cbfb-MYH11-ΔHABD* Knock-In mice and characterization of leukemia developed in these mice.

(A) Schematic representation of the generation of conditional *Cbfb-MYH11-ΔHABD* knock-in mice using CRISPR technology. (B) Western blot analysis detecting the expression of CBFβ-SMMHC or CBFβ-SMMHC-ΔHABD in Cytoplasmic (Cy) and nuclear (Nu) Fractions of the bone marrow from the indicated mice, 2–3 weeks post-pIpC treatment. GAPDH and Lamin B were used as internal control for cytoplasmic and nuclear protein, respectively. (C) Bar graphs showing the white blood cells (WBC), Hematocrit and platelets in the end stage primary mice. n = 7-9 per group. (D-E) Dot plot showing the percentages of B cells ($CD19^+$), T cells ($CD3^+$, $CD4^+$, $CD8^+$), myeloid cells ($Gr1^+$, $Mac1^+$) and immature cells ($c\text{-}Kit^+$) in the peripheral blood, spleen and bone marrow of the end stage primary mice showed in Figure 1C (D, n = 7-10 per group) and transplanted mice showed in Figure 1F (E, n = 11-12 per group). All data in panels C, D and E are shown in mean ± SEM, with each dot representing an individual sample. $^{\#}P < .05$, each compared with the $Mx1\text{-}CreCbfb^{+/56M}$ mice. Statistical significance was assessed using 2-tailed Student's t test.

Supplemental Figure 3

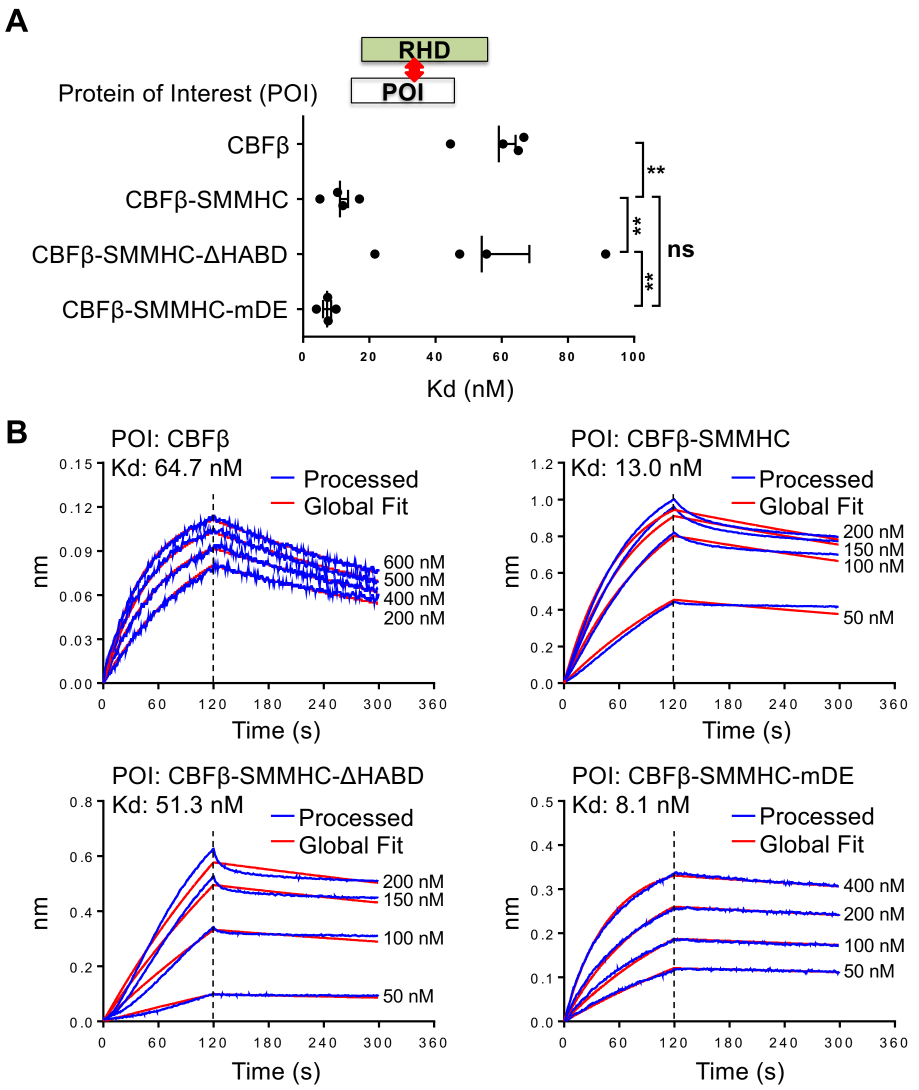

Supplemental Figure 3: Binding affinity of CBFβ and CBFβ-SMMHC variants for the RHD (RUNX1).

(A-B) BLI assay was conducted to evaluate the interaction between the RHD and indicated proteins of interest (POI). (A) Dot plot showing the binding affinity (mean ± SEM), with each dot representing an individual replicate. ns: no significant and ***P* < *.001*. Statistical significance was assessed using1-way ANOVA followed by Tukey's post hoc test. (B) Representative binding curves from the BLI assay, showing both the association and dissociation phases.

Supplemental Figure 4

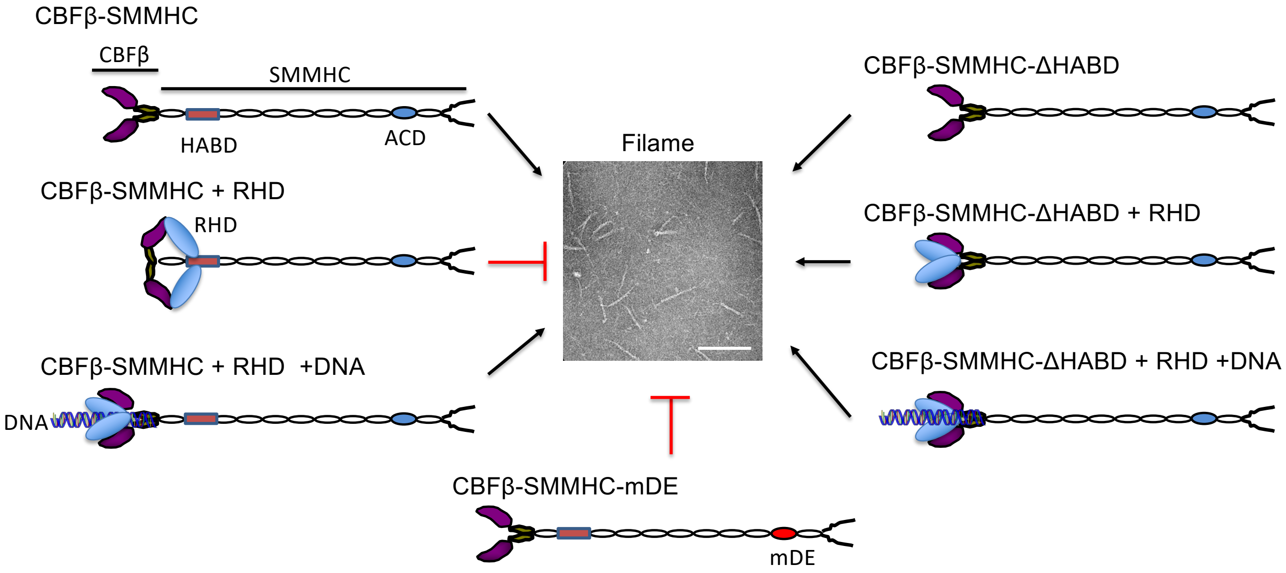

Supplemental Figure 4: Diagram illustrating a model to explain the effects of CBFβ-SMMHC mutations, RHD and target DNA on filament formation (Figure 2D).

The binding of RHD to the HABD domain potentially interferes with interactions between CBFβ-SMMHC molecules through the SMMHC tail. The addition of target DNA oligonucleotides may compete with HABD for RHD binding, which takes RHD away from HABD and restores CBFβ-SMMHC filament formation. Deletion of the HABD domain renders CBFβ-SMMHC resistant to the RHD inhibition of filament formation. Finally, mutations in the ACD domain (mDE) inhibits CBFβ-SMMHC's ability to form dimers, and consequently they cannot form filaments. In summary, certain mutations or interactions alter the structural integrity of the SMMHC region, potentially disrupting its ability to form filaments.

Supplemental Figure 5

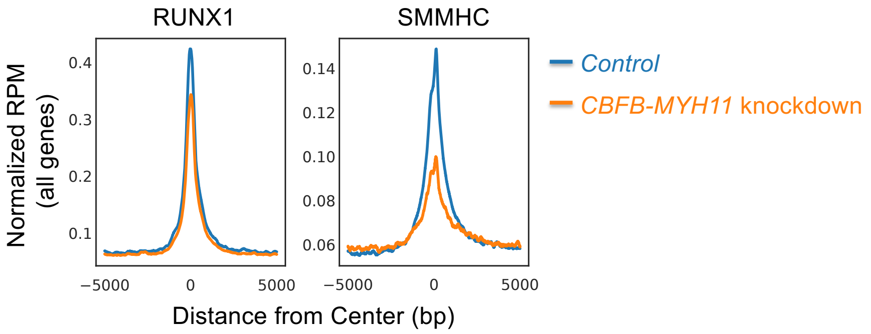

Supplemental Figure 5: RUNX1 binding enrichment at TSSs was reduced following *CBFB-MYH11* knockdown in ME-1 cells

Reanalysis of ChIP-seq data for RUNX1 and SMMHC in ME-1 cells following *CBFB-MYH11* knockdown, as reported in reference 32. Shown are aggregated average ChIP-seq binding profiles of the indicated proteins at the TSSs of all genes, normalized to reads per million (RPM). The center of each plot corresponds to the TSS.

Supplemental Figure 6

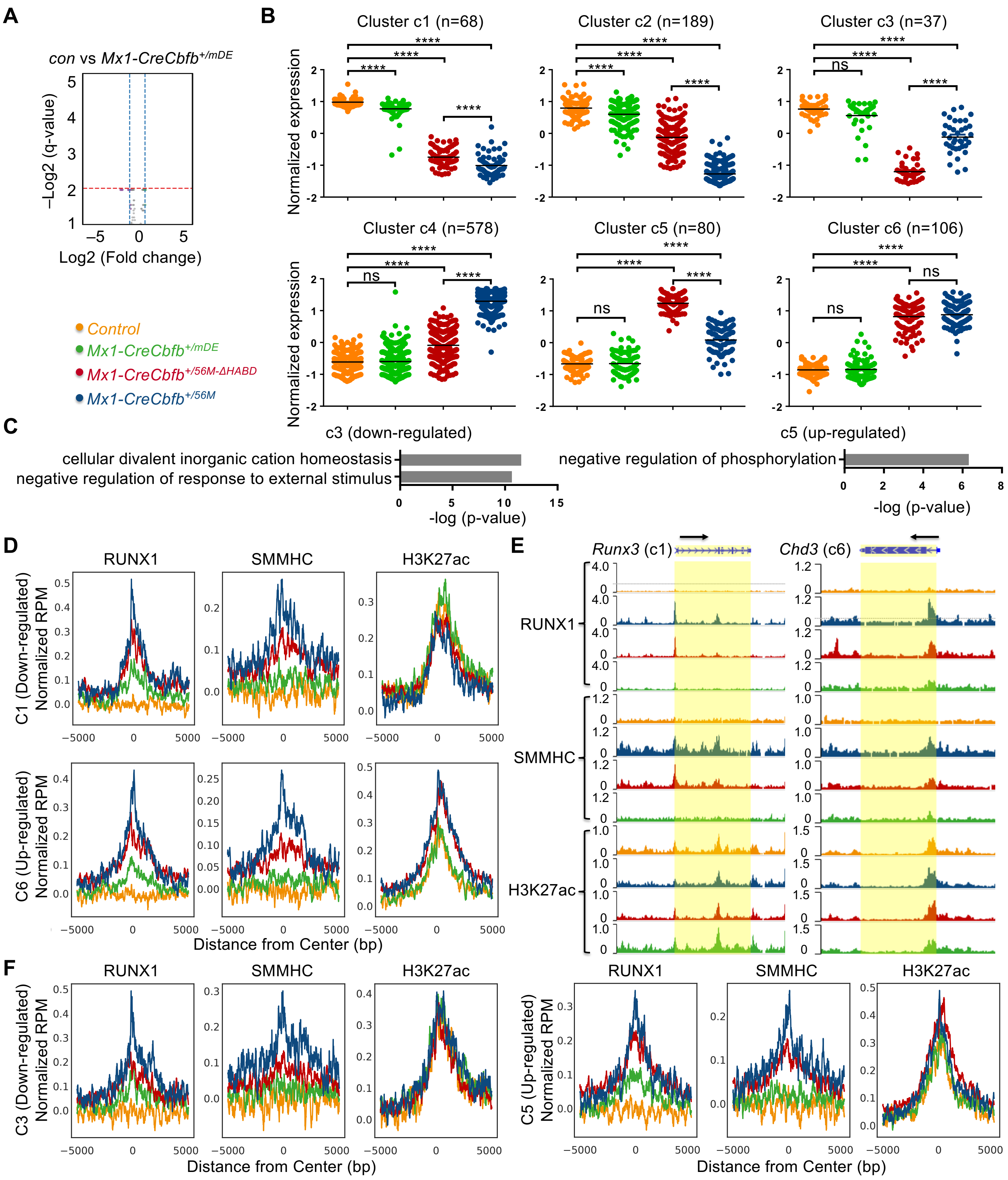

Supplemental Figure 6: RUNX1/CBFβ-SMMHC directly regulates inflammation-associated genes and CBFA2T3 target genes.

(A-C) RNA-seq was performed on LK cells isolated from control, *Mx1-CreCbfb*$^{+/56M}$, *Mx1-CreCbfb*$^{+/56M\text{-}\Delta HABD}$ and *Mx1-CreCbfb*$^{+/mDE}$ mice 2 to 3 weeks after pIpC treatment. (A) Volcano Plot to show there were no DEGs between control and *Mx1-CreCbfb*$^{+/mDE}$ mice. (B) Normalized expression levels of genes in clusters c1, c2, c3, c4, c5, and c6 (mean ± SEM), as shown in Figure 6B. Each dot representing a gene. ns: no significant and ****$P < .0001$. Statistical significance was assessed using1-way ANOVA followed by Tukey's post hoc test. (C) Gene ontology analysis of the DEGs in clusters c3 and c5, as shown in Figure 4B. Left panel, only terms with -log (p-value) > 10 are shown; Right panel, terms with -log (p-value) > 5 are shown. (D) Average binding profiles of the indicated proteins at the TSSs of genes in clusters c1 and c6, respectively. Normalized with IgG signals subtracted for each condition. The center locations in the graphs (0) are the TSSs. (E) Genome browser images displaying RUNX1, SMMHC, and H3K27ac binding at the *Runx3* (cluster c1) and *Chd3* (cluster c6) genes are shown, with the gene direction indicated by black arrows. ChIC-seq tracks are color-coded to match the corresponding mouse conditions shown in B. (F) Average binding profiles of the indicated proteins at the TSSs of genes in clusters c3 and c5, respectively. Normalized with IgG signals subtracted for each condition. The center locations in the graphs (0) are the TSSs.

Supplemental Figure 7

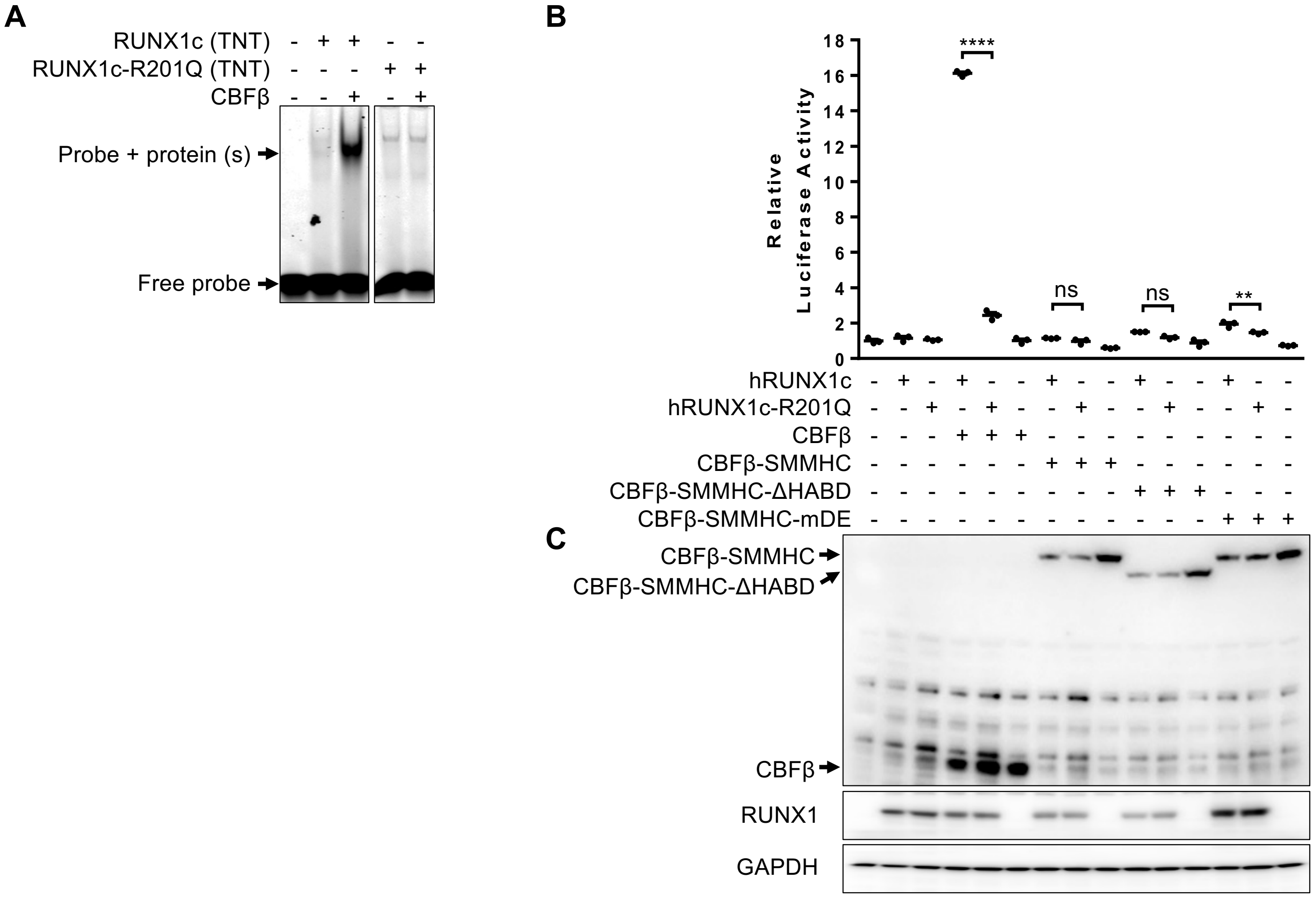

Supplemental Figure 7: RUNX1-R201Q mutant lacks DNA binding affinity and fails to transactivate *CSF1R* luciferase activity.

(A) Electrophoretic Mobility Shift Assay showed that RUNX1c-R201Q is unable to interact with target DNA oligo. (B-C) Luciferase reporter assay in 293T cells transfected with a *CSF1R* promoter-driven luciferase reporter plasmid and plasmids encoding the indicated proteins. (B) Reporter assay are shown (mean ± SEM), with each dot representing an individual replicate. $^{**}P < .01$; $^{****}P < .0001$ and ns: no significant. Statistical significance was assessed using1-way ANOVA followed by Tukey's post hoc test. (C) Representative expression levels of the transfected proteins for this reporter assay by western blot analysis.

Supplemental Figure 8

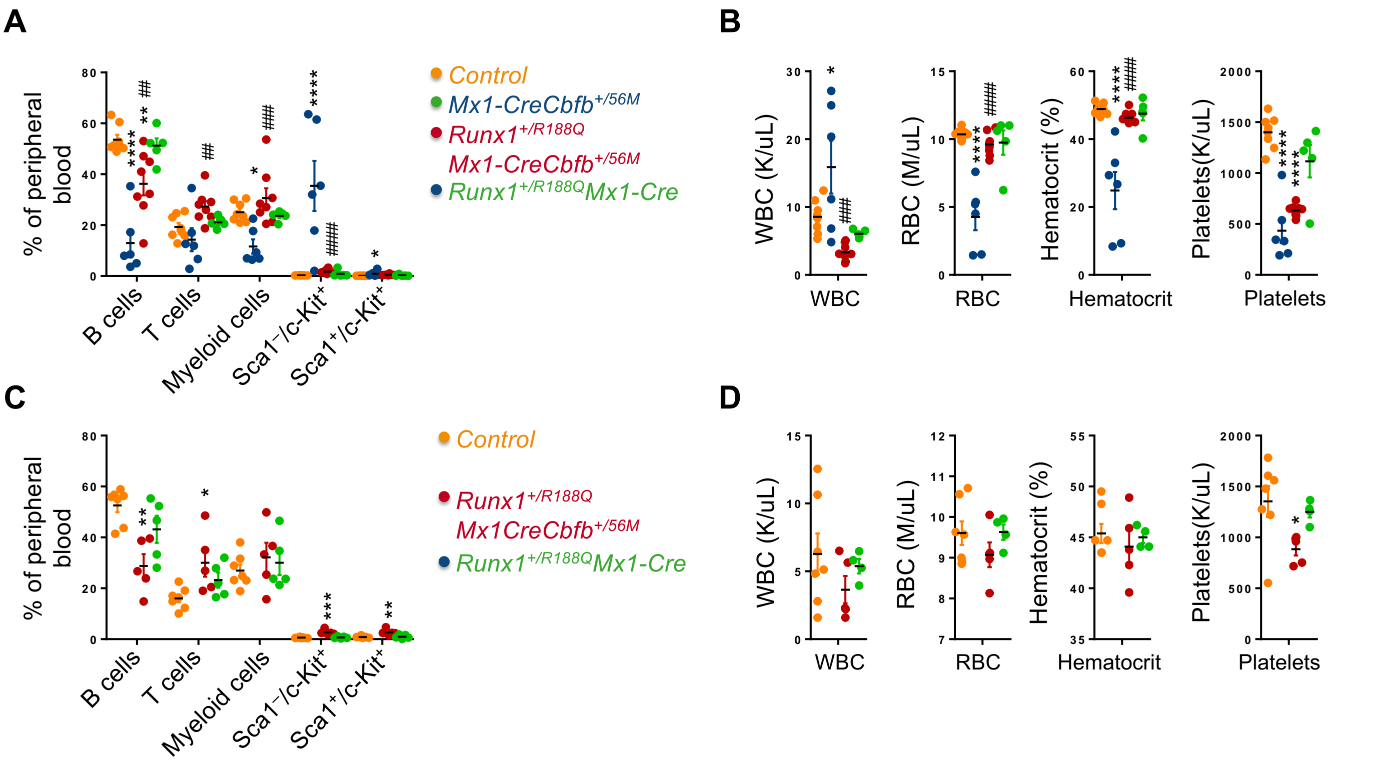

Supplemental Figure 8: Leukemia like phenotypes were observed in *Mx1-CreCbfb*$^{+/56M}$ mice but not in *Runx1*$^{+/R188Q}$*Mx1-CreCbfb*$^{+/56M}$.

(A-D) Dot plot showing the percentages of B cells (CD19+), T cells (CD3+, CD4+, CD8^{+}), myeloid cells (Gr1+, Mac1^{+}), immature cells (c-Kit^{+}), as well as white blood count (WBC), red blood count (RBC), Hematocrit, and Platelet levels in the peripheral blood of the indicated mice 11 weeks (A-B, n = 5-8 per genotype) or 12 months (C-D, n = 4-7 per genotype) after pIpC treatment (mean ± SEM). Each dot representing an individual sample. Data are represented as mean ± SEM, *$P < .05$; **$P < .01$; ***$P < .001$; ****$P < .0001$, each compared with the control. ##$P < .01$; ###$P < .001$; ####$P < .0001$, each compared with the *Mx1-CreCbfb*$^{+/56M}$ group. Statistical significance was assessed using1-way ANOVA followed by Tukey's post hoc test.

Supplemental Figure 9

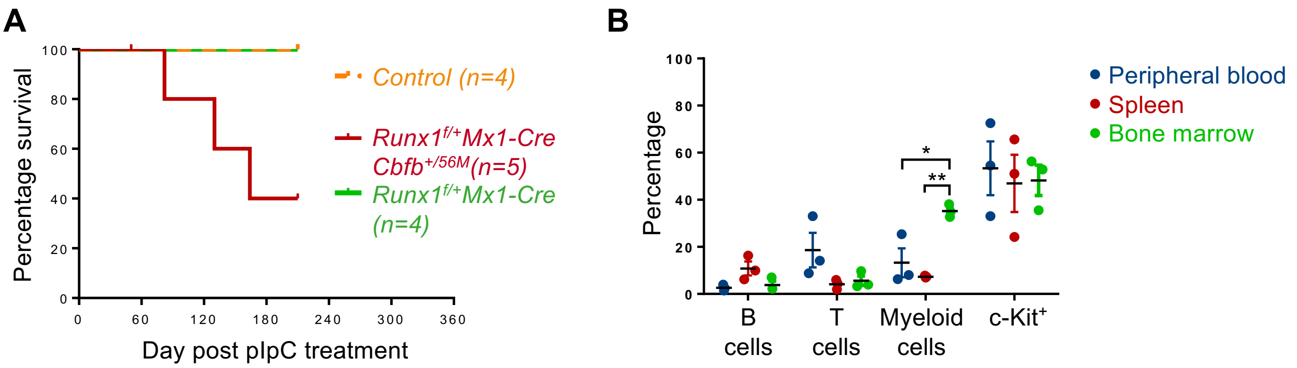

Supplemental Figure 9: Leukemia developed in *Runx1$^{f/+}$Mx1-CreCbfb$^{+/56M}$* mice.

(A-B) Mice of indicated genotypes were treated with pIpC to induce the expression of *Cbfb-MYH11* and knock out of one *Runx1* allele. (A) Kaplan-Meier survival curves of these mice are shown. (B) Dot plot showing the percentages (mean ± SEM) of cell populations in the peripheral blood, spleen and bone marrow of end-stage leukemic *Runx1$^{f/+}$Mx1-CreCbfb$^{+/56M}$* mice (n = 3), with each dot representing an individual sample. Data are represented as mean ± SEM, *$P < .05$ and **$P < .01$. Statistical significance was assessed using1-way ANOVA followed by Tukey's post hoc test.

Supplemental Figure 10

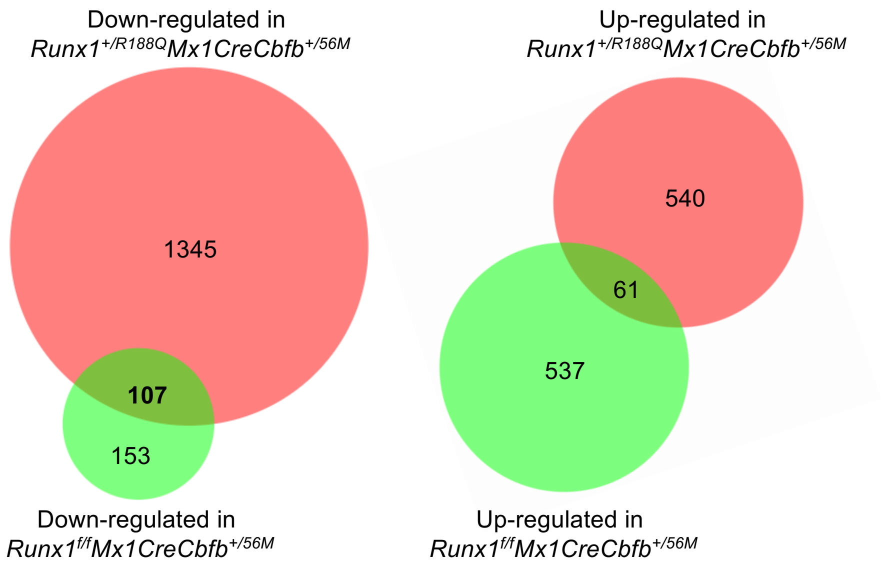

Supplemental Figure 10: *Runx1$^{+/R188Q}$* and *Runx1$^{-/-}$* exhibit distinct effects on the transcriptome changes induced by *Cbfb-MYH11*.

Venn diagrams showing the overlaps of DEGs in AMP cells between *Mx1-CreCbfb$^{+/56M}$* mice and either *Runx1$^{+/R188Q}$Mx1CreCbfb$^{+/56M}$* or *Runx1$^{f/f}$Mx1-CreCbfb$^{+/56M}$* mice. Down-regulated DEGs and up-regulated DEGs displayed separately.

Supplemental Figure 11

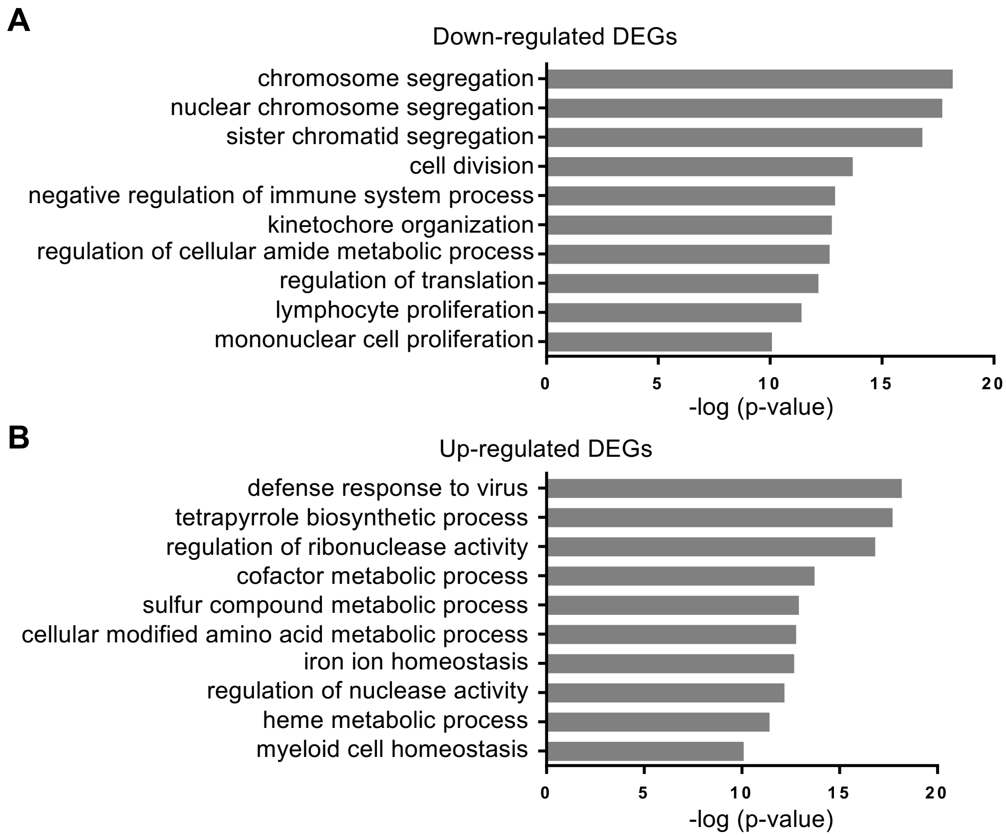

Supplemental Figure 11: Geno ontology analysis of the differentially expressed genes in AMP cells between *Mx1-CreCbfb*$^{+/56M}$ mice and *Runx1*$^{+/R188Q}$*Mx1CreCbfb*$^{+/56M}$ mice based on RNA-seq

Gene ontology analysis of the down-regulated DEGs (A) and up-regulated DEGs shown in Figure 6B. Terms with -log (p-value) > 10 are shown.

Supplemental Figure 12

A

	Mx1-CreCbfb $^{+/56M}$		*Runx1* $^{+/R188Q}$ *Mx1-CreCbfb* $^{+/56M}$	
	Repeat 1	Repeat 2	Repeat 1	Repeat 2
Estimated Number of Cells	15111	11272	14567	11959
Mean Reads per Cell	10969	16963	12620	15523
Median Genes per Cell	2207	2816	2565	2964
Number of Reads	165752001	191202676	183837699	185633832
Valid Barcodes	96.90%	96.40%	96.00%	96.10%
Sequencing Saturation	27.70%	31.10%	25.90%	25.00%
Q30 Bases in Barcode	96.10%	96.20%	96.20%	96.10%
Q30 Bases in RNA Read	93.50%	94.00%	94.10%	93.80%
Q30 Bases in UMI	95.30%	95.20%	95.20%	95.20%
Reads Mapped to Genome	94.20%	95.30%	94.30%	94.30%
Reads Mapped Confidently to Genome	91.10%	92.20%	91.10%	91.10%
Reads Mapped Confidently to Intergenic Regions	1.50%	1.50%	1.60%	1.60%
Reads Mapped Confidently to Intronic Regions	13.10%	13.60%	12.00%	13.20%
Reads Mapped Confidently to Exonic Regions	76.50%	77.10%	77.50%	76.30%
Reads Mapped Confidently to	83.90%	84.70%	83.80%	83.30%
Reads Mapped Antisense to Gene	5.30%	5.50%	5.30%	5.80%
Fraction Reads in Cells	97.00%	95.70%	96.10%	96.20%
Total Genes Detected	21789	21113	21612	20853
Median UMI Counts per Cell	5614	8004	7081	8566

B

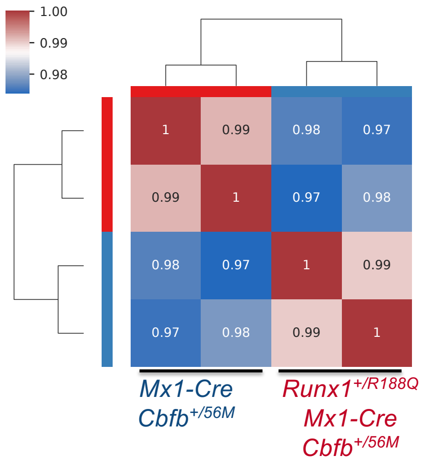

C

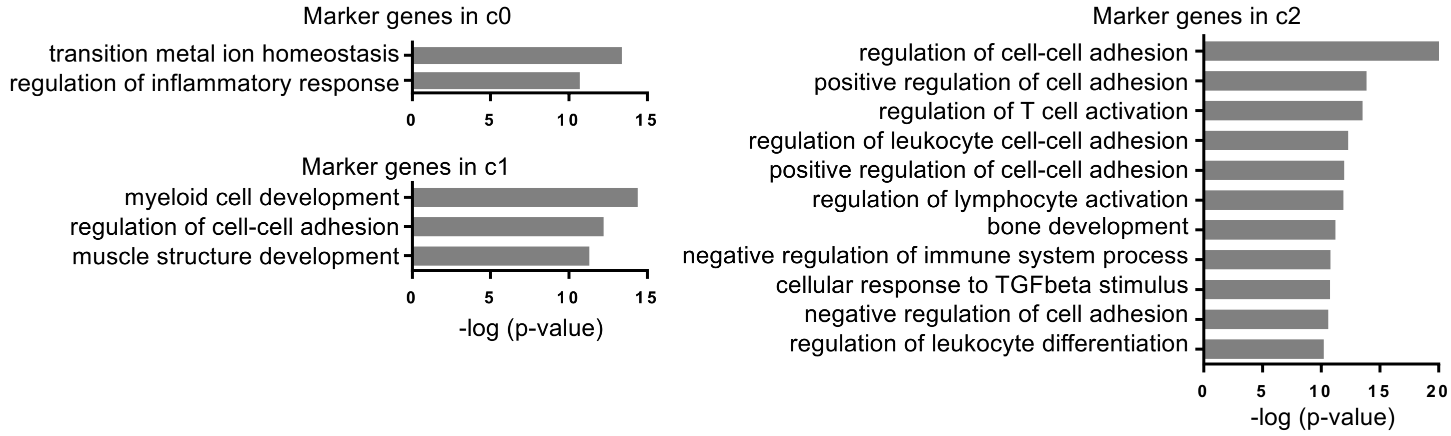

Supplemental Figure 12: Bioinformatic analysis of scRNA-RNA seq data from the AMP population.

(A) Statistics of the scRNA-seq data. (B) Heatmap displaying the technical variations between the replicates. (C) Gene ontology analysis of marker genes identified in cluster c0, c1 and c2 as described in Figure 7D. Terms with -log (p-value) > 10 are shown.
