# Supplementary material for: CBFβ-SMMHC–driven leukemogenesis requires enhanced RUNX1-DNA binding affinity in mice
Source: J Clin Invest. 2025 Aug 5;135(19):e192923. doi: 10.1172/JCI192923 (PMC12483565; doi:10.1172/JCI192923)
Supplement: Unedited blot and gel images [file jci-135-192923-s111.pdf]

Full unedited gel for Figure S2B_CBFβ

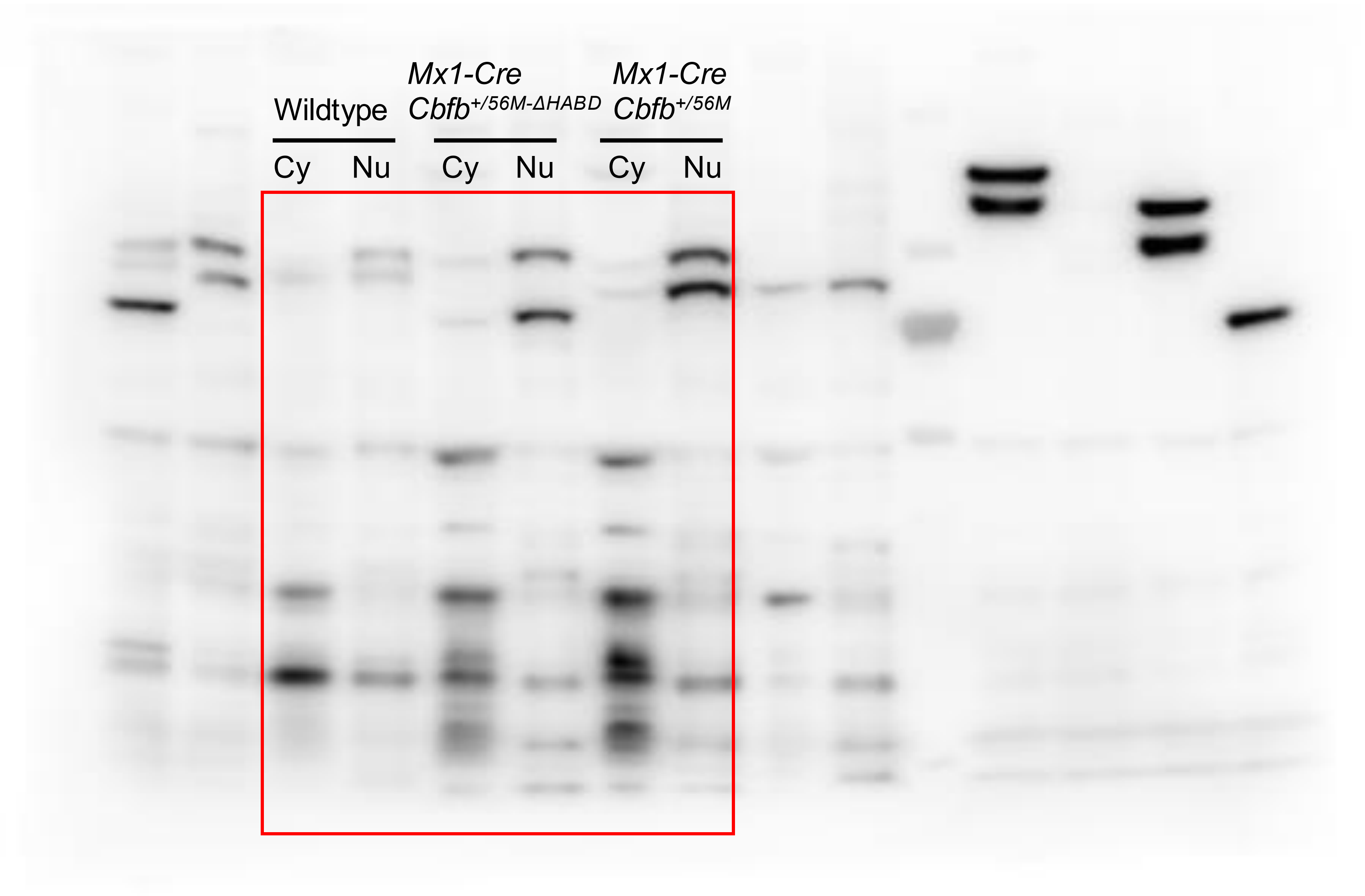

Full unedited gel for Figure S2B_GAPDH

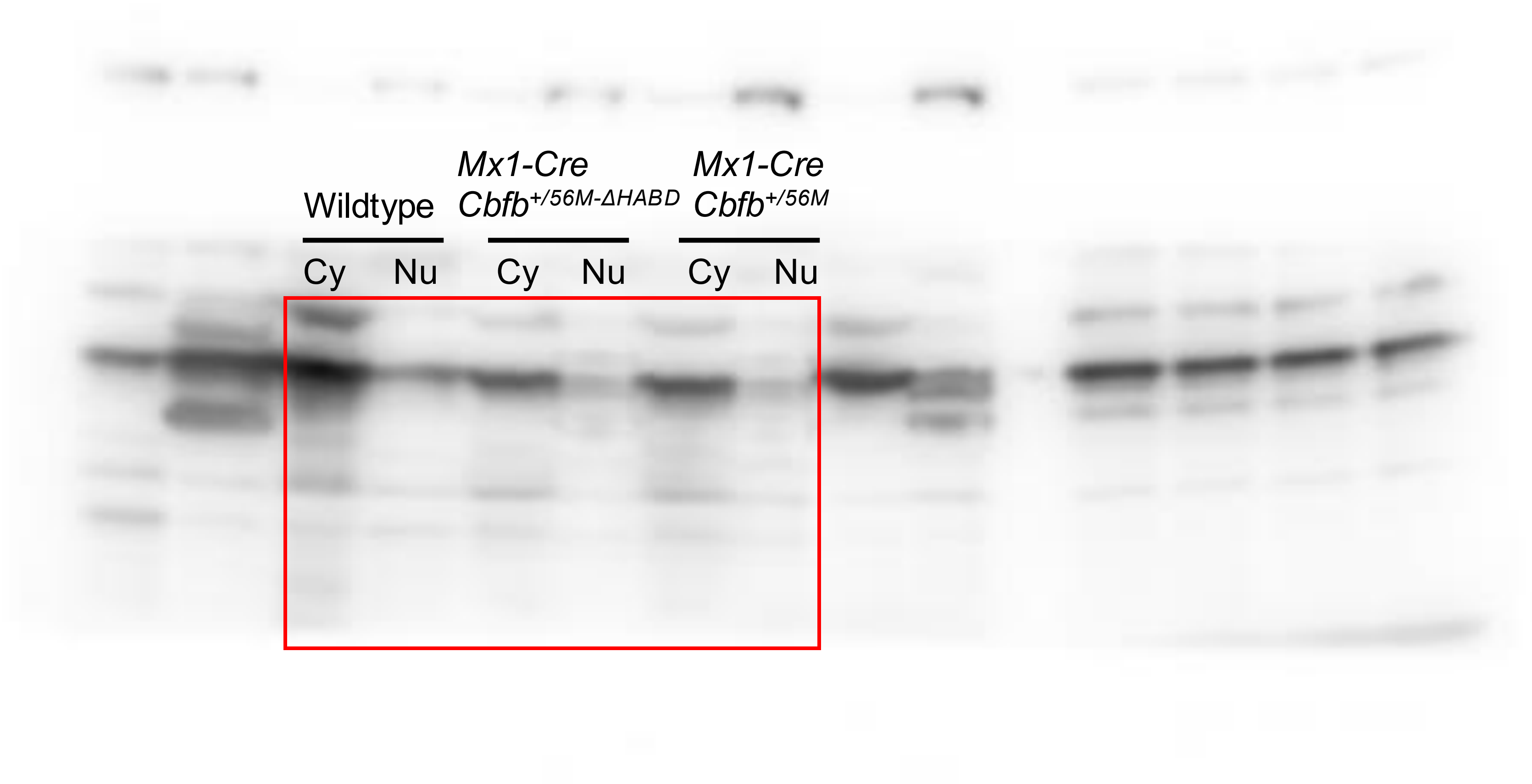

Full unedited gel for Figure S2B_Lamin B

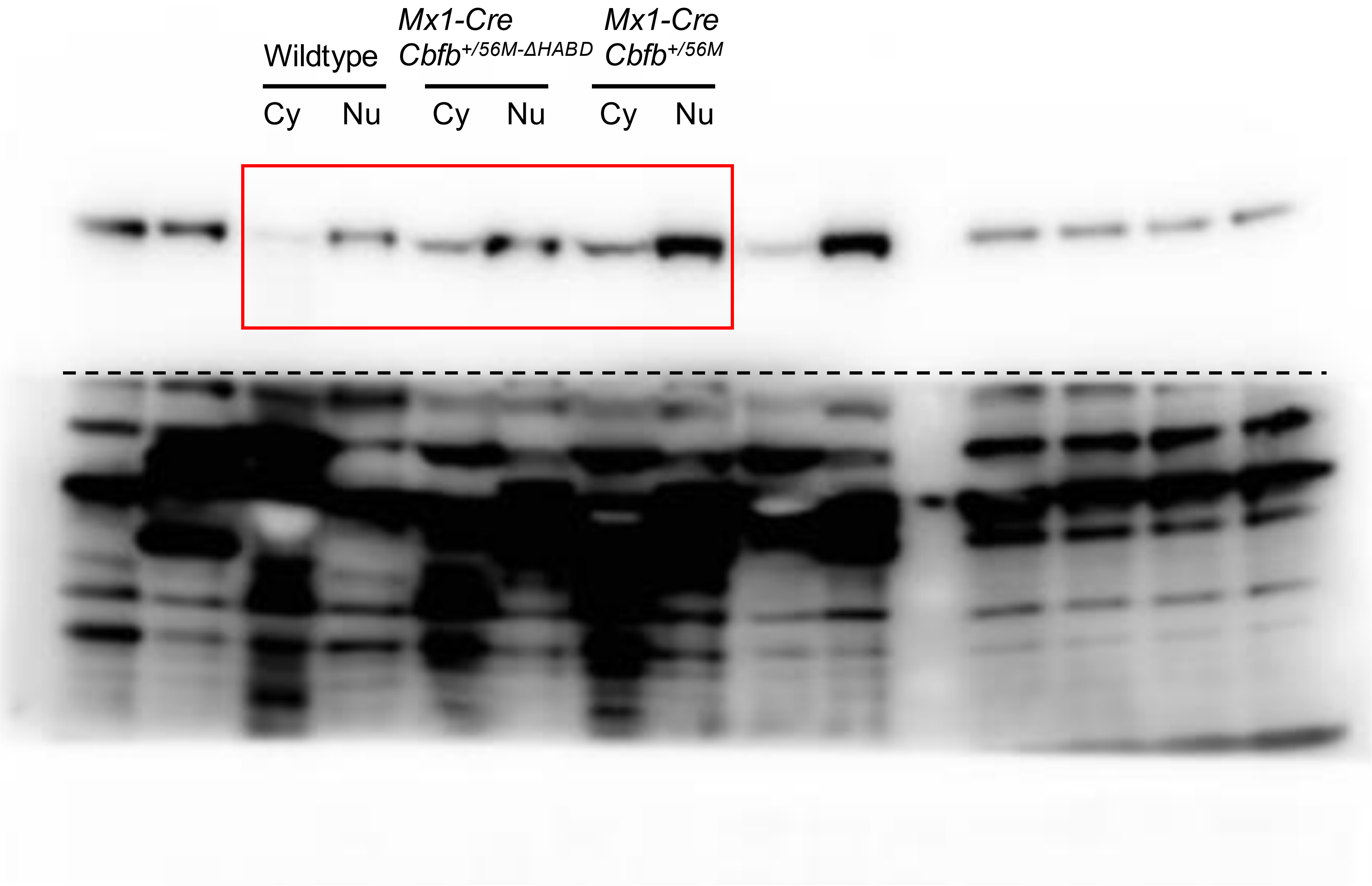

Membrane was cut to blot with different antibodies

Full unedited gel for Figure S7A

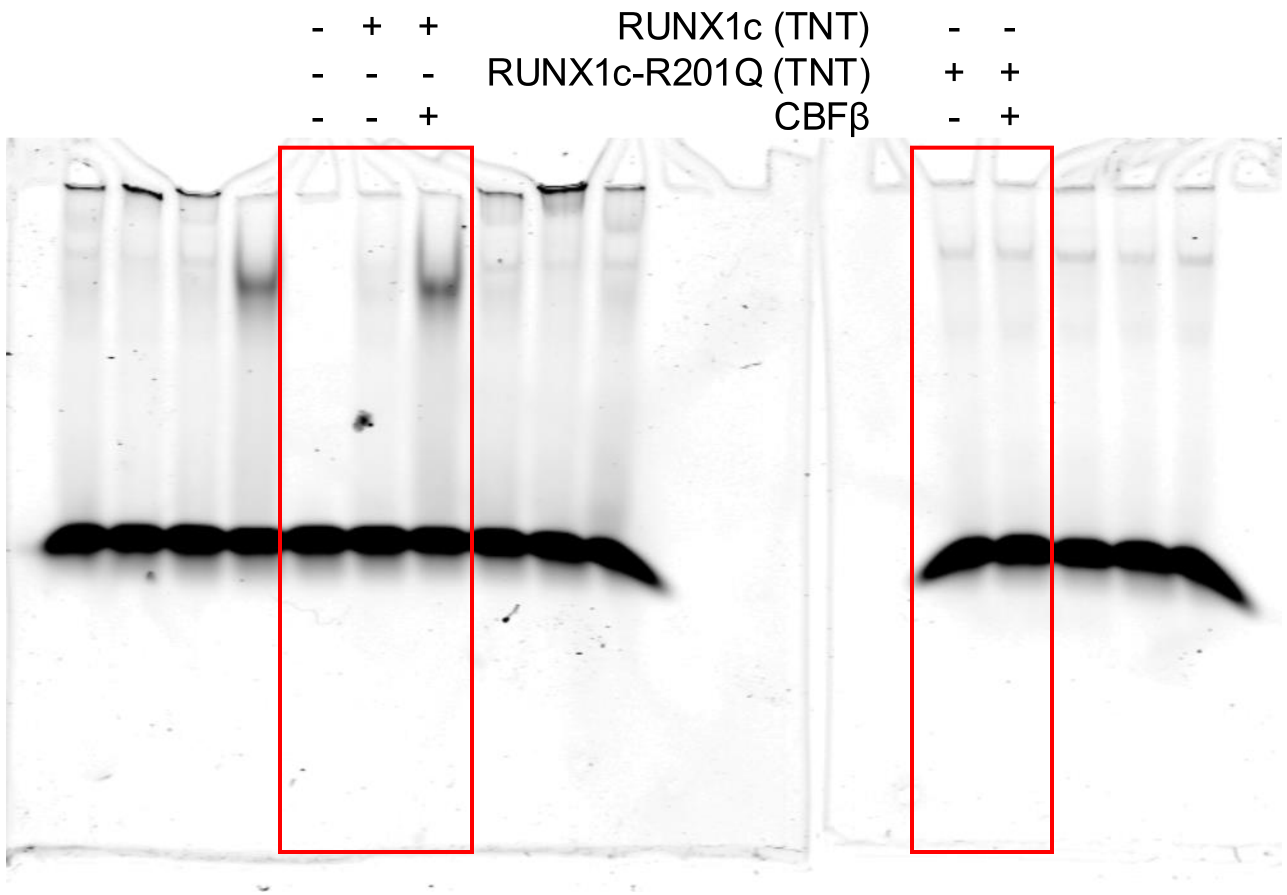

Full unedited gel for Figure S7B_top panel

hRUNX1c	-	+	-	+	-	-	+	-	-	+	-	-	+	-	-	hRUNX1c
hRUNX1c-R201Q	-	-	+	-	+	-	-	+	-	-	+	-	-	+	-	hRUNX1c-R201Q
CBFβ	-	-	-	+	+	+	-	-	-	-	-	-	-	-	-	CBFβ
CBFβ-SMMHC	-	-	-	-	-	-	+	+	+	-	-	-	-	-	-	CBFβ-SMMHC
CBFβ-SMMHC-ΔHABD	-	-	-	-	-	-	-	-	-	+	+	+	-	-	-	CBFβ-SMMHC-ΔHABD
CBFβ-SMMHC-mDE	-	-	-	-	-	-	-	-	-	-	-	-	+	+	+	CBFβ-SMMHC-mDE

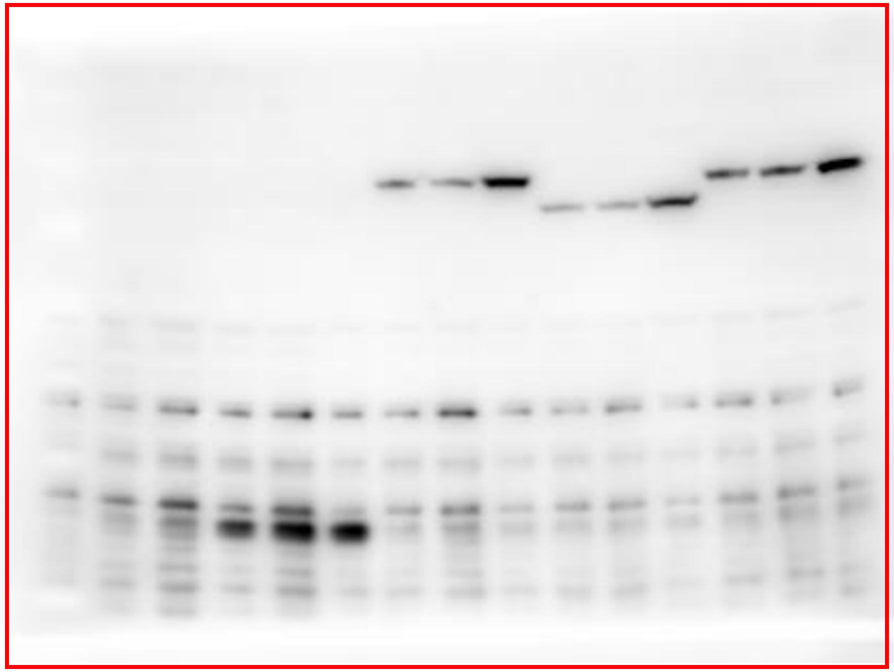

Full unedited gel for Figure S7B_RUNX1_GAPDH

hRUNX1c	-	+	-	+	-	-	+	-	-	+	-	-	+	-	-
hRUNX1c-R201Q	-	-	+	-	+	-	-	+	-	-	+	-	-	+	-
CBFβ	-	-	-	+	+	+	-	-	-	-	-	-	-	-	-
CBFβ-SMMHC	-	-	-	-	-	-	+	+	+	-	-	-	-	-	-
CBFβ-SMMHC-ΔHABD	-	-	-	-	-	-	-	-	-	+	+	+	-	-	-
CBFβ-SMMHC-mDE	-	-	-	-	-	-	-	-	-	-	-	-	+	+	+

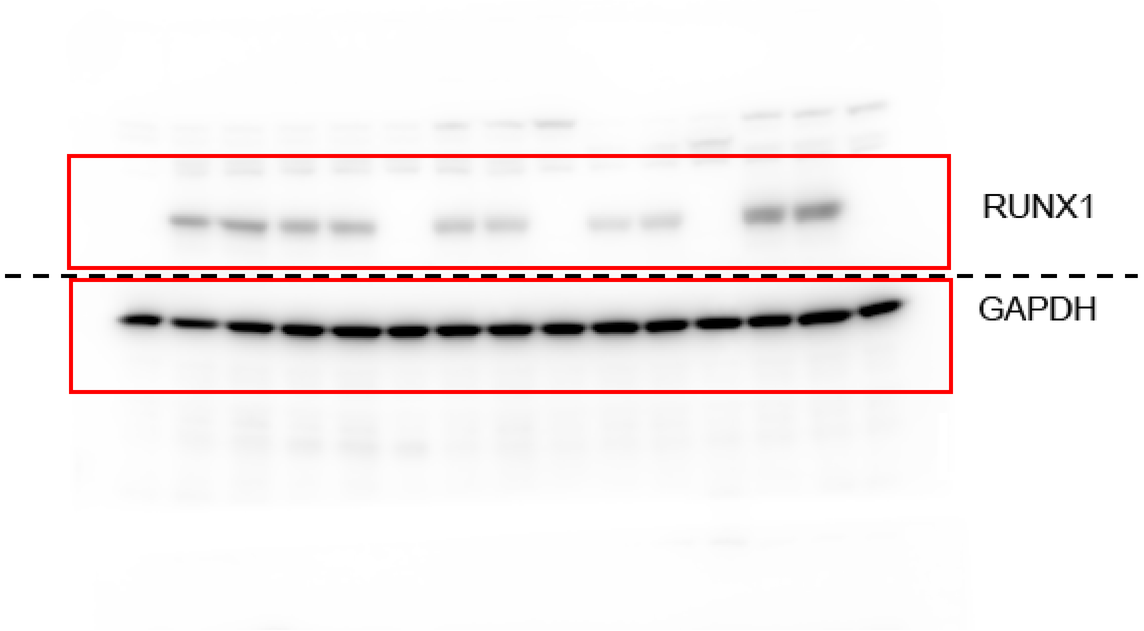

Membrane was cut to blot with different antibodies
